# *XAP5 CIRCADIAN TIMEKEEPER* coordinates circadian rhythms and anthocyanin biosynthesis independently of splicing

**DOI:** 10.1093/plphys/kiag235

**Published:** 2026-04-22

**Authors:** Hongtao Zhang, Stacey L Harmer

**Affiliations:** Department of Plant Biology, College of Biological Sciences, University of California, Davis, CA 95616, USA; Department of Plant Biology, College of Biological Sciences, University of California, Davis, CA 95616, USA

## Abstract

Circadian clocks provide plants with an adaptive advantage by enabling them to anticipate daily environmental changes. The periodicity of circadian clocks is regulated at multiple levels of gene expression, including transcription, mRNA processing, translation, and protein modification. Numerous mRNA splicing factors have been implicated in maintaining circadian period length. However, these factors often play additional roles in transcription, making it difficult to determine whether they affect the clock through splicing-dependent or splicing-independent mechanisms. We and others have shown that XAP5 CIRCADIAN TIMEKEEPER (XCT) and components of the PRE-MRNA-PROCESSING FACTOR 19 (PRP19) complex, including the functionally redundant PRP19A and PRP19B, physically associate and regulate both splicing and circadian rhythms. Here, our transcriptome analyses reveal that the antagonistic regulation of circadian period length by XCT and PRP19 likely occurs through splicing-independent mechanisms. Interestingly, both factors co-regulate expression of a substantial set of shared target genes involved in RNA metabolism, photosynthesis, and stress responses despite having largely distinct targets for splicing. Gene co-expression analysis followed by functional characterization identified anthocyanin biosynthesis as another process antagonistically regulated by XCT and PRP19. Nonetheless, we found genetic perturbation of anthocyanin production does not affect circadian period, suggesting that the observed correlation between anthocyanin levels and circadian period may instead reflect disruption of a shared upstream regulatory pathway. Together, our findings suggest involvement of XCT and PRP19 in the transcriptional coordination of anthocyanin biosynthesis and biological timing, expanding their known roles beyond mRNA splicing.

## Teaser

Two evolutionarily conserved RNA-processing proteins oppositely control anthocyanin accumulation and circadian rhythms through mechanisms beyond RNA splicing.

## Graphical Abstract

**Graphical Abstract:**
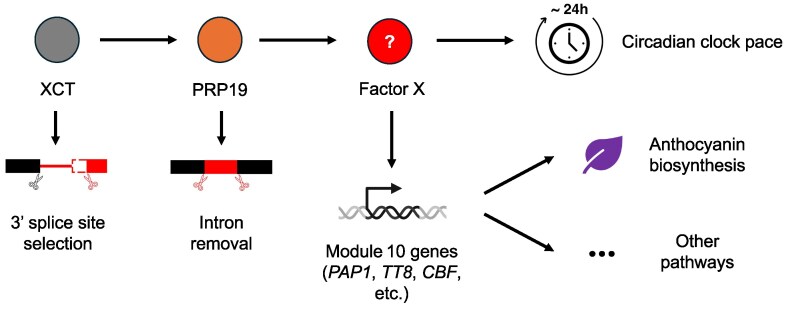

## Introduction

Daily rhythms are widely observed across living organisms. In plants, various physiological and molecular processes, such as organ growth, metabolism, stress responses, gene expression, and protein modification, exhibit time-of-day dependent activities (Greenham and McClung 2015; Harmer 2025). Although these rhythms are often synchronized to daily environmental cycles, many are governed by an internal timekeeping mechanism known as the circadian clock (Harmer 2009). This endogenous clock enhances plant fitness by allowing anticipation of daily environmental changes (Dodd et al. 2005; Troein et al. 2009).

While the composition and number of core circadian components vary across the green lineage, plant circadian clocks are primarily driven by interlocked transcriptional–translational feedback loops (Creux and Harmer 2019). For example, the morning-expressed transcription factors CIRCADIAN CLOCK-ASSOCIATED1 (CCA1) and LATE ELONGATED HYPOCOTYL (LHY) repress expression of the afternoon and early night-phased genes *PSEUDO-RESPONSE REGULATOR 7* (*PRR7*) and *TIMING OF CAB EXPRESSION 1* (*TOC1*). Their gene products in turn repress the expression of *CCA1*, *LHY*, and other transcriptional activators, such as *LIGHT-INDUCIBLE AND CLOCK-REGULATED 2* (*LNK2*) (Nohales and Kay 2016). In addition to transcriptional control, post-transcriptional and post-translational mechanisms add important layers of regulation, enhancing the robustness and precision of circadian rhythms (Hernando et al. 2017; Harmer 2025).

Extensive research has revealed multiple connections between RNA processing and circadian clock function in plants (Staiger and Brown 2013; Shang et al. 2017; Harmer 2025). In eukaryotes, precursor messenger RNAs (pre-mRNAs) often contain introns that need to be removed before nuclear export and translation (Matera and Wang 2014). This process, known as pre-mRNA splicing, is carried out through the dynamic stepwise assembly and disassembly of the spliceosome, a large ribonucleoprotein complex (Shi 2017). Noncanonical selection of 5′ and/or 3′ splice sites can generate alternatively spliced mRNA isoforms. These may lead to nonsense-mediated decay of the mRNAs or production of proteins with altered functions (Staiger and Brown 2013). As a result, alternative splicing plays important roles in numerous biological processes, including circadian rhythms. To date, many splicing factors have been shown to impact circadian period length (Hong et al. 2010; Sanchez et al. 2010; Jones et al. 2012; Wang et al. 2012; Perez-Santángelo et al. 2014; Schlaen et al. 2015; Marshall et al. 2016; Feke et al. 2019). Mutations of some of these factors cause alternative splicing of known clock genes. However, in many cases, the observed alternative splicing events do not fully account for the circadian phenotypes of the mutants (Jones et al. 2012; Wang et al. 2012; Perez-Santángelo et al. 2014; Schlaen et al. 2015; Zhang et al. 2023).

**Figure 1:**
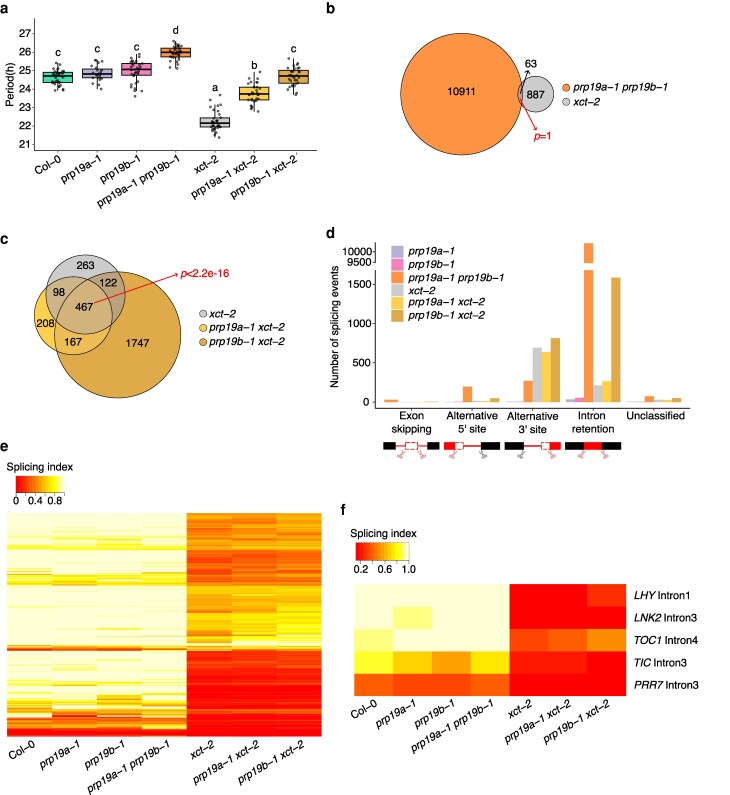
Loss of *PRP19* function rescues the circadian clock but not the transcriptome-wide splicing defects of *xct-2*. a) Circadian period of wild-type Col-0 and loss-of-function mutants for *XCT* and *PRP19*. Period length was estimated by luciferase activity of a *CCR2*::*LUC+* reporter assayed in plants maintained under constant temperature and light conditions (n = 23 to 36). The upper edge, middle line, and lower edge in the boxplot represent the 75% quartile, median, and 25% quartile of the data, respectively. Different letters indicate genotypes with significantly different period estimates as determined by linear regression with genotype as a fixed effect followed by Tukey's post hoc test for multiple comparisons (*P* < 0.05). Experiments were independently repeated 3 times with similar results (Zhang et al. 2023). b) Limited overlap between statistically significant differential splicing events identified by RNA-seq in *prp19a-1 prp19b-1* and *xct-2* mutants compared to Col-0. Statistical significance of overlapping events was determined using 1-tailed Fisher's exact test. c) Significant overlap between differential splicing events in *xct-2*, *prp19a-1 xct-2*, and *prp19b-1 xct-2* compared to Col-0. The 467 *xct-2*-induced differential splicing events that are also present in both *prp19a-1 xct-2* and *prp19b-1 xct-2* were defined as overlapping events and analyzed for statistical significance using 1-tailed Fisher's exact test. d) Distribution of different types of differential splicing events in the corresponding mutants compared to Col-0. e) Splicing indices for all of the alternative 3′ splice site events identified in *xct-2* relative to Col-0 in the indicated genotypes. Each row represents a specific splicing event. Splicing index values were calculated using PSI and PIR. A lower value indicates higher usage of an alternative 3′ splice site. f) Splicing indices for the splicing events of core circadian clock genes that are significantly altered in *xct-2* relative to Col-0 in the indicated genotypes. A lower splicing index value corresponds to increased usage of an alternative 3′ splice site (for *LHY*, *LNK2*, *TIC*, and *TOC1*) or decreased intron retention (for *PRR7*).

Recent studies suggest that splicing factors may in some cases influence the circadian clock through splicing-independent mechanisms. We and others have identified an Arabidopsis (*Arabidopsis thaliana*) gene, *XAP5 CIRCADIAN TIMEKEEPER* (*XCT*), that functions in both pre-mRNA splicing and circadian clock regulation (Martin-Tryon and Harmer 2008; Liu et al. 2022; Zhang et al. 2023). *xct* mutants exhibit a shortened circadian period and global defects in 3′ splice site selection. Interestingly, we found that loss-of-function mutations in either of the 2 core subunits of the PRE-MRNA-PROCESSING FACTOR 19 (PRP19, also known as MOS4-ASSOCIATED or MAC in plants) complex, PRP19A or PRP19B, suppress the short-period phenotype of *xct-2* (Zhang et al. 2023; reproduced in Fig. 1a). Components of the PRP19 complex physically associate with XCT and are implicated in splicing, circadian clock regulation, and many other fundamental biological processes (Jia et al. 2017, 2023; Feke et al. 2019; Liu et al. 2022; Zhang et al. 2023). Surprisingly, reverse transcription quantitative PCR (RT-qPCR) revealed that the splicing defects of 5 core clock genes that are differentially spliced in *xct* mutants, *LHY*, *LNK2*, *TOC1*, *TIME FOR COFFEE* (*TIC*), and *PRR7*, are not restored by loss of PRP19 function (Zhang et al. 2023). These results suggest that perturbed splicing of these clock genes does not mediate XCT's effect on circadian period. However, it remains unknown whether XCT and PRP19 influence the clock through the splicing of other genes or by splicing-independent mechanisms.

Pre-mRNA splicing often occurs co-transcriptionally (Bentley 2005; Moore and Proudfoot 2009). Accordingly, splicing factor proteins often carry out additional nuclear functions, such as regulating transcription and chromatin state (Moore and Proudfoot 2009; Tognacca et al. 2020). For example, certain SERINE/ARGININE-RICH (SR) proteins are recruited by RNA Polymerase II (Pol II) to coordinate transcriptional elongation and splicing fidelity (Das et al. 2007; Long and Caceres 2009). In plants, many splicing-related proteins directly associate with RNA Pol II and regulate the transcription of both microRNAs (miRNAs) and protein-coding genes (Hirayama and Shinozaki 1996; Zhang et al. 2013; Dolata et al. 2015; Li et al. 2016, 2019; Wang et al. 2019; Xin et al. 2024, 2025). Intriguingly, several of these proteins are core components of or associate with the PRP19 complex, raising the possibility that PRP19 and its interacting protein XCT may also play splicing-independent roles in transcriptional regulation.

Although it remains unclear whether PRP19 and XCT directly modulate transcription in Arabidopsis, cross-lineage evidence supports such roles. In yeast and humans, homologs of PRP19 have been shown to participate directly in transcriptional initiation and elongation (Chanarat et al. 2011; Dwyer et al. 2026). Similarly, XCT homologs in fission yeast, green algae, and mice function as chromatin-binding transcription factors (Anver et al. 2014; Li et al. 2018a; Wang et al. 2025). Consistent with these findings, a recent study shows that Arabidopsis PRP19A physically associates with chromatin (Jiang et al. 2023), suggesting a possible direct role in transcription. In addition, epitope-tagged PRP19B and XCT have been found to co-purify with transcription-related proteins, including RNA Pol II subunits and members of the RNA POLYMERASE II-ASSOCIATED FACTOR 1 (PAF1) complex (Jia et al. 2023; Gao et al. 2024). Given their high sequence similarity and evolutionarily conserved roles in splicing (Martin-Tryon and Harmer 2008; Monaghan et al. 2009; Chanarat and Sträßer 2013; Lee et al. 2020), it is plausible that PRP19 and XCT also contribute to transcriptional regulation of gene expression in Arabidopsis.

*XCT* and *PRP19* are both pleiotropic genes. Independent studies of *xct* and *prp19a prp19b* mutants have revealed that both genes are involved in a wide range of biological processes, including small RNA biogenesis, immune responses, light signaling, and hypocotyl elongation (Martin-Tryon and Harmer 2008; Monaghan et al. 2009; Ellison et al. 2011; Fang et al. 2015; Jia et al. 2017; Li et al. 2018b; Kumimoto et al. 2021; Jiang et al. 2023) in addition to their roles in mRNA splicing and circadian clock regulation (Jia et al. 2017, 2023; Feke et al. 2019; Zhang et al. 2023). Notably, several of these phenotypes have been associated with reduced occupancy of transcriptional regulators at specific gene targets (Fang et al. 2015; Jiang et al. 2023). However, it remains unclear whether XCT and PRP19 contribute to these processes by regulating a common set of genome-wide targets and whether their effects are mediated through splicing-dependent or splicing-independent mechanisms.

Given the nonadditive genetic interaction between *XCT* and *PRP19* in regulating circadian period length (Zhang et al. 2023; reproduced in Fig. 1a) and their functional overlap in other biological processes, we hypothesized that shared molecular mechanisms may underlie their broader regulatory roles. To test this, we combined transcriptome profiling with genetic approaches to investigate how mRNA splicing and gene expression contribute to XCT and PRP19 function. Genome-wide splicing analysis revealed that XCT and PRP19 proteins play distinct roles in splicing and regulate circadian period through splicing-independent mechanisms. A direct comparison of differentially expressed and differentially spliced genes in the *xct-2* mutant indicates that XCT influences gene expression beyond its role in splicing. Gene co-expression analyses of single and double mutants for *XCT* and *PRP19* uncovered a significant set of shared target genes involved in RNA metabolism, photosynthesis, and stress responses. In addition to circadian period, anthocyanin biosynthetic gene expression and pigment accumulation are also antagonistically regulated by XCT and PRP19, perhaps through the same genetic pathway. However, we show that anthocyanin accumulation itself does not influence circadian period length. This suggests that a shared upstream transcriptional mechanism controlled antagonistically by XCT and PRP19 may simultaneously regulate both anthocyanin biosynthesis and circadian rhythms. Together, our results suggest that XCT and PRP19 coordinate anthocyanin production and circadian function through splicing-independent roles.

## Results

### Normal *PRP19* function is essential for the effects of *XCT* on the circadian clock but not pre-mRNA splicing

Given that XCT and PRP19 proteins co-purify with many spliceosomal components and both affect genome-wide pre-mRNA splicing patterns (Jia et al. 2017; Liu et al. 2022; Zhang et al. 2023), we hypothesized that their antagonistic regulation of circadian period length might rely on their functions in splicing. To test this, we performed high read-depth RNA sequencing (RNA-seq) to assess genome-wide splicing patterns. Previous studies have shown that both transcription and alternative splicing of a large proportion of the *Arabidopsis* genome are under circadian regulation (Covington et al. 2008; Romanowski et al. 2020; Yang et al. 2020). Consequently, collecting samples at a single time point may result in false positives in the identification of differentially expressed or spliced genes, as apparent differences in transcript levels may actually reflect circadian phase differences between samples (Hsu and Harmer 2012). To mitigate this issue, we grew Arabidopsis seedlings in constant light and then harvested them at 2 h intervals over a 22-, 24-, or 26-h period (covering a full circadian cycle for each of the different genotypes as described in Fig. 1a) and then pooled equal amounts of tissue from each time point into a single RNA-seq library (Fig. S1). Although this approach may lead to reduced detection of time-of-day-specific differences in gene expression or splicing between genotypes, it eliminates the confounding effects of delayed circadian phase in *prp19a-1 prp19b-1* (Feke et al. 2019) and advanced phase in *xct-2* mutants (Martin-Tryon and Harmer 2008) that would have been observed had we harvested at a single time point.

Across the 21 RNA-seq libraries (7 genotypes with 3 biological replicates), we obtained an average of 37.4 million paired-end short reads uniquely mapped to the Arabidopsis genome (Table S1). Using these reads, we performed genome-wide splicing analysis to identify statistically significant differential splicing of exon–intron junctions in each mutant relative to Col-0 (hereafter referred to as differential splicing events). Single loss-of-function mutants *prp19a-1* and *prp19b-1* exhibit only minor disruptions to global splicing patterns (Fig. S2), consistent with their reported redundancy in regulating the circadian clock and plant immunity (Monaghan et al. 2009; Zhang et al. 2023). In contrast, the *prp19a-1 prp19b-1* double mutant displays 10,974 differential splicing events compared to Col-0 (Fig. 1b). Among the 950 differential splicing events detected in *xct-2*, only 63 overlaps with those in *prp19a-1 prp19b-1*, a significantly lower overlap than expected by chance (1-tailed Fisher's exact test, *P* = 1). Classification of differential splicing events revealed that *xct-2* mainly disrupts 3′ splice site selection, while *prp19a-1 prp19b-1* predominantly causes intron retention (Fig. 1d; Dataset S1), consistent with previous reports conducted separately on each mutant (Jia et al. 2017; Liu et al. 2022; Zhang et al. 2023). Furthermore, Gene Ontology (GO) enrichment analysis showed that genes mis-spliced in *xct-2* and *prp19a-1 prp19b-1* encode proteins that act in different cellular compartments (Fig. S3). Together, these results indicate that XCT and PRP19 primarily affect distinct aspects of pre-mRNA splicing.

We next asked whether *prp19a-1 xct-2* and *prp19b-1 xct-2* double mutants could suppress the splicing defects observed in *xct-2*. Despite the restored circadian period (Fig. 1a), most of the differential splicing events in *xct-2* are retained in the double mutants (Fig. 1c). Of the 950 events identified in *xct-2*, 467 persist in both *prp19a-1 xct-2* and *prp19b-1 xct-2*, representing a significantly enriched overlap (1-tailed Fisher's exact test, *P* < 2.2e–16). The number of alternative 3′ splice site events, the predominant type of splicing defect in *xct-2*, remains similar among *xct-2*, *prp19a-1 xct-2*, and *prp19b-1 xct-2* mutants (Fig. 1d). To evaluate whether the extent of these alternative 3′ splice site defects is alleviated in the double mutants, we examined the splicing index at each affected intron. A lower splicing index value indicates higher usage of an alternative 3′ splice site. The indices are similarly reduced among *xct-2*, *prp19a-1 xct-2*, and *prp19b-1 xct-2* (Fig. 1e; Dataset S1), indicating no alleviation of *xct-2* splicing defects in the double mutants. Together, these findings demonstrate that the major genome-wide splicing defects observed in *xct-2* are not rescued in either *prp19a-1 xct-2* or *prp19b-1 xct-2*.

Intriguingly, *prp19b-1 xct-2*, but not *prp19a-1 xct-2*, displays a notable increase in intron retention events when compared to the single mutants (Fig. 1d). The intron retention pattern observed in *prp19b-1 xct-2* closely resembles that seen in *prp19a-1 prp19b-1* (Fig. S4). Notably, neither the *prp19b-1* nor *xct-2* single mutant shows comparable intron retention, suggesting a synergistic role for XCT and PRP19B in maintaining splicing fidelity in addition to XCT's previously described, independent function in 3′ splice site selection.

Precisely regulated transcription and splicing of core circadian clock genes is crucial for the maintenance of normal circadian period (Harmer 2025). We and others found that introns in a handful of core clock genes are differentially spliced in *xct-2* mutants (Fig. 1f; Liu et al. 2022; Zhang et al 2023). Given the restoration of circadian period in *prp19 xct-2* double mutants, we compared the splicing indices of affected introns in these genes. Consistent with our genome-wide analysis, the reduced splicing indices of core clock genes are comparable among *xct-2*, *prp19a-1 xct-2*, and *prp19b-1 xct-2* (Fig. 1f). This result is also in agreement with our previous RT-qPCR results obtained at the expression peak of the examined genes (Zhang et al. 2023). We also analyzed splicing patterns across 1,717 previously reported Arabidopsis transcription factors (Jin et al. 2017). Among the 32 transcription factors that are differentially spliced in *xct-2* compared to Col-0, none show rescued splicing indices in *prp19 xct-2* double mutants (Fig. S5). This finding suggests that the restored circadian period in the double mutants is unlikely to result from reversal of splicing defects in a transcription factor. Altogether, our data indicate that neither global nor core clock gene-specific splicing defects in *xct-2* are rescued by loss of PRP19 function. This suggests that XCT and PRP19 regulate circadian timing and mRNA splicing through genetically separable pathways.

### *XCT* regulates gene expression in addition to splicing

**Figure 2:**
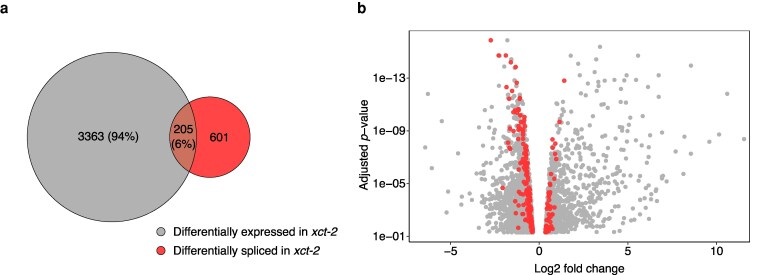
*XCT* controls gene expression in addition to splicing. a) A significant proportion (94%) of DEGs are not differentially spliced in *xct-2* compared to Col-0. DEGs were determined using a fold-change cutoff of 1.2 and an adjusted *P* value threshold of 0.05. b) Log2 fold changes of gene expression and their corresponding adjusted *P* values (Benjamini and Hochberg correction) for all significantly DEGs in *xct-2* compared to Col-0. Red dots represent the 205 genes that are both significantly differentially spliced and differentially expressed in *xct-2*, whereas gray dots indicate the 3,363 DEGs that are not differentially spliced.

Homologs of XCT exhibit high sequence conservation across eukaryotes (Martin-Tryon and Harmer 2008), suggesting they may retain conserved biochemical functions. Multiple XCT homologs have been directly implicated in the control of transcription (Anver et al. 2014; Li et al. 2018a; Wang et al. 2025). Interestingly, a recent immunoprecipitation-mass spectrometry study reported that Arabidopsis XCT co-purified with transcription-associated proteins, including components of RNA Pol II (Gao et al. 2024). Therefore, we investigated whether Arabidopsis XCT regulates gene expression independently of its role in splicing. Differential gene expression analysis of RNA-seq data identified 3,568 significantly differentially expressed genes (DEGs) in *xct-2* compared to Col-0 (Fig. 2a; Datasets S2 and S3). Strikingly, 3,363 (94%) of these DEGs do not exhibit differential splicing in *xct-2*. Moreover, genes with the largest changes in transcript abundance, especially those with >4-fold upregulation or <0.25-fold downregulation, are not differentially spliced (Fig. 2b). These results indicate that Arabidopsis XCT may contribute to regulation of gene expression independently of its role in splicing.

To explore the biological processes associated with gene expression changes in *xct-2*, we conducted GO analysis. Among the 1,469 upregulated genes in *xct-2*, GO terms related to RNA splicing and RNA helicase activity are significantly enriched (Fig. S6a and b), implying that XCT also transcriptionally impacts the splicing machinery. Genes involved in UV response pathways are also upregulated in *xct-2*, in line with previous findings showing hypersensitivity of *xct-2* mutants to UV radiation and DNA damage (Kumimoto et al. 2021). Additionally, GO terms associated with flavonoid biosynthesis and isoprenoid binding are enriched among upregulated genes (Fig. S6a and b), pointing to a potential role for XCT in regulating these metabolic pathways. In contrast, the 2,099 downregulated genes in *xct-2* are predominantly enriched for processes related to photosynthesis and responses to cellular oxygen levels (Fig. S6c and d), in agreement with earlier reports of reduced chlorophyll content and chloroplast developmental defects in *xct-2* mutants (Martin-Tryon and Harmer 2008; Gao et al. 2024). Therefore, our results suggest that transcriptomic changes are likely linked to many of the physiological and developmental defects previously reported in *xct-2*.

Our prior work in fission yeast *Schizosaccharomyces pombe* showed that Xap5, an ortholog of Arabidopsis XCT, is highly enriched at transposable element loci and that *Δxap5* mutants exhibit significant upregulation of these elements (Anver et al. 2014). We therefore investigated whether Arabidopsis XCT similarly regulates transposable element expression. Of the 236 transposable elements detected in our RNA-seq data, 97 are differentially expressed in *xct-2*, representing a highly significant enrichment (1-tailed Fisher's exact test, *P* < 2.2e–16; Fig. S7a). Furthermore, overall normalized expression of transposable elements is significantly elevated in *xct-2* compared to Col-0 (Fig. S7b). These findings support an evolutionarily conserved role for XCT in the silencing of transposons. However, upregulation of transposable elements is not suppressed in either *prp19 xct-2* double mutant (Fig. S7b), indicating that these changes are unlikely to underlie the rescued circadian period observed in the double mutants.

### Differential gene expression analysis indicates that *XCT* and *PRP19* regulate common targets

Similar to XCT, PRP19 homologs in other species have been shown to directly participate in transcriptional regulation (Chanarat and Sträßer 2013). A recent study demonstrated that Arabidopsis PRP19A also binds DNA to modulate target gene expression (Jiang et al. 2023). However, a direct comparison of gene expression regulated by XCT and PRP19 has not previously been reported. We first assessed the overall variation in the transcriptomes of Col-0, *xct-2*, and *prp19* mutants using principal component analysis (PCA). Along the first 2 principal components (PCs), which together explains 62% of the transcriptomic variance, both *xct-2* and the *prp19a-1 prp19b-1* double mutant display strong separation from Col-0 (Fig. S8a). Interestingly, these 2 mutants cluster closely on PC1, but are clearly separated on PC2 and PC3 (Fig. S8b). This pattern indicates that XCT and PRP19 share overlapping as well as distinct influences on global gene expression. Notably, the *prp19a-1 xct-2* and *prp19b-1 xct-2* double mutants are nearly indistinguishable from *xct-2* on PC1 and PC2 (Fig. S8a). However, on PC3 their expression profiles cluster more closely with Col-0 than with *xct-2* (Fig. S8b). Considering that these double mutants rescue the circadian period defect of *xct-2* without restoring splicing or morphological phenotypes (Fig. 1; Zhang et al. 2023), our PCA results suggest that changes in a relatively small subset of genes correlate with the rescue of clock function. Together, these analyses indicate that both XCT and PRP19 strongly impact gene expression and that some of this regulation likely occurs through shared molecular pathways.

**Figure 3:**
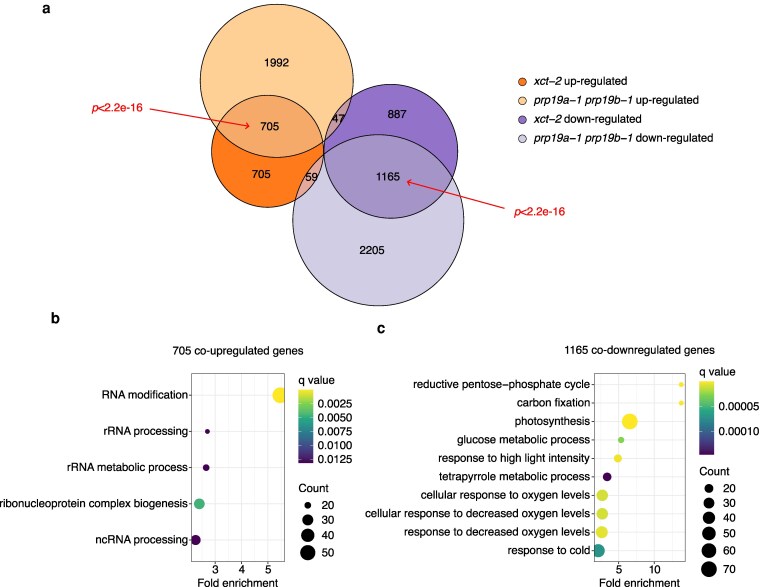
*XCT* and *PRP19* promote and inhibit expression of common target genes. a) Commonly and uniquely up- and downregulated genes in *xct-2* and *prp19a-1 prp19b-1* compared to Col-0. Statistical significance of overlapping genes was determined using 1-tailed Fisher's exact test. b to c) Biological processes over-represented in the 705 genes that are co-upregulated (b) and the 1,165 genes that are co-downregulated (c) in *xct-2* and *prp19a-1 prp19b-1* relative to all 20,258 expressed genes as determined by GO analysis.

To identify genes co-regulated by XCT and PRP19, we compared DEGs in *xct-2* and *prp19a-1 prp19b-1* mutants relative to Col-0. Among the 3,568 DEGs in *xct-2* and 6,173 DEGs in *prp19a-1 prp19b-1*, 705 genes are co-upregulated and 1,165 are co-downregulated, both representing highly significant overlaps (1-tailed Fisher's exact test, *P* < 2.2e–16; Fig. 3a; Dataset S2). The 705 co-upregulated genes are significantly enriched for biological processes related to RNA metabolism, including modification of ribosomal RNA (rRNA) and noncoding RNA (ncRNA) (Fig. 3b). These results, combined with established roles for XCT and PRP19 in mRNA splicing and small RNA biogenesis (Fang et al. 2015; Jia et al. 2017; Zhang et al. 2023), suggest that both proteins contribute broadly to RNA metabolism. Co-downregulated genes are enriched for functions in photosynthesis and cellular responses to low oxygen (Fig. 3c). Although direct involvement of the PRP19 in photosynthesis has not been revealed before, a study reported downregulation of photosynthetic genes in plants mutant for *MAC7*, another PRP19 complex gene (Jia 2017), suggesting that PRP19 proteins may play a similar role as XCT in positive regulation of photosynthetic gene expression. Altogether, our RNA-seq analyses suggest that XCT and PRP19 collaboratively regulate gene expression programs involved in RNA metabolism and photosynthesis.

### WGCNA reveals XCT and PRP19 affect both shared and distinct pathways

**Figure 4:**
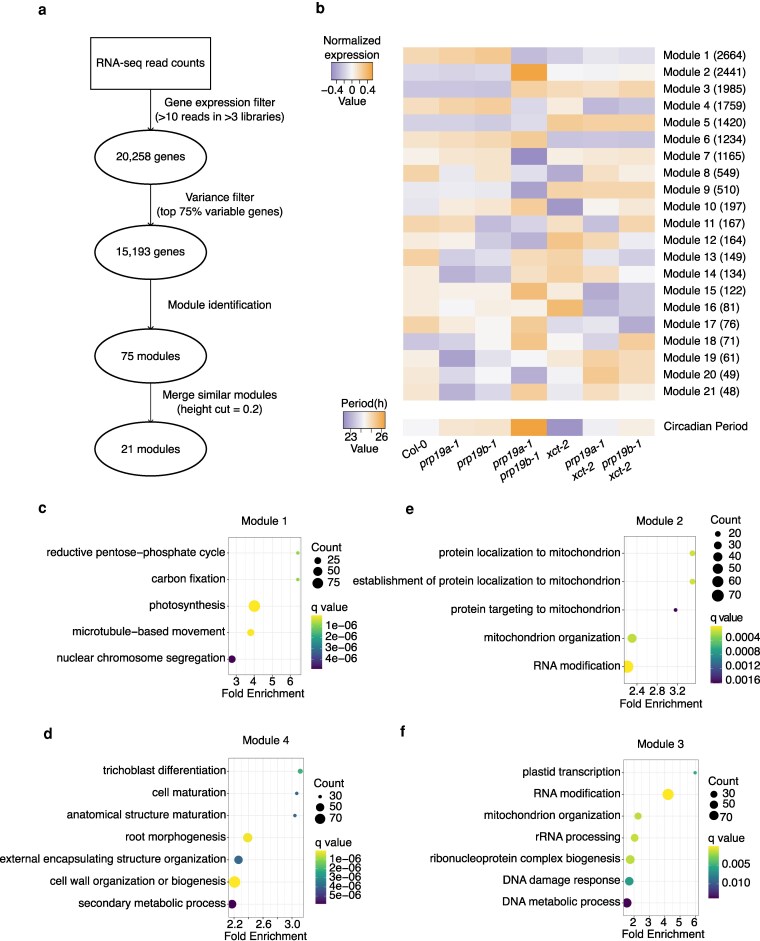
Gene co-expression analysis reveals processes both commonly and uniquely regulated by *XCT* and *PRP19*. a) Simplified workflow for WGCNA. b) Top heatmap: normalized expression of 21 module eigengenes identified by WGCNA across the indicated genotypes. Each row corresponds to a module eigengene, labeled with the module name and the number of genes in that module. Bottom heatmap: mean circadian period for the corresponding genotypes, as shown in Fig. 1a. c to f) Biological processes over-represented among the genes in Modules 1, 4, 2, and 3 relative to all 20,258 expressed genes as determined by GO analysis.

Our PCA results demonstrate that *xct-2*, *prp19a-1 prp19b-1*, and the *prp19 xct-2* double mutants have both overlapping and distinct transcriptomic profiles (Fig. S8). Thus, to comprehensively examine gene expression differences across these genotypes, we performed weighted gene co-expression network analysis (WGCNA) (Fig. 4a). We first filtered out uninformative genes by excluding those with low or minimally variable expression across samples. After initial clustering of co-expressed gene modules, those with highly similar eigengenes, defined as the first PC of gene expression within each module, were merged. With these approaches, we identified 21 co-expression modules, ranging in size from 48 to 2,664 genes (Fig. 4b; Dataset S4).

Consistent with our pairwise differential gene expression results (Fig. 3), the 4 largest modules consist of genes commonly up- or downregulated in both *prp19a-1 prp19b-1* and *xct-2* relative to Col-0 (Fig. 4b). These genes are further separated into distinct modules based on differences in magnitude of expression changes across all the mutants. For example, although genes in Modules 1 and 4 are generally downregulated in *prp19a-1 prp19b-1*, *xct-2*, and *prp19 xct-2* double mutants, those in Module 4 are less reduced in *xct-2* than in the other mutants (Fig. 4b; Fig. S9a and b). These distinct expression patterns correspond to different biological processes: Module 1 genes are enriched for photosynthesis and microtubule-associated processes (Fig. 4c; Dataset S5), whereas Module 4 genes are enriched for root development and cell wall organization (Fig. 4d). A recent study reported that PRP19A and PRP19B promote lateral root emergence by degradation of a key transcription factor (Yu et al. 2024). Therefore, the repression of Module 4 genes in *prp19a-1 prp19b-1* may indicate a direct role for PRP19 in regulating root development, while XCT may influence this process indirectly by modulating PRP19 function.

A similar pattern is observed for Modules 2 and 3. Both modules contain genes upregulated in *prp19a-1 prp19b-1*, *xct-2*, and the *prp19 xct-2* double mutants relative to Col-0. However, Module 2 genes are more strongly induced in *prp19a-1 prp19b-1*, while those in Module 3 show uniform induction across the 4 mutants (Fig. 4b; Fig. S9c and d). Both modules are enriched for genes involved in RNA modification and mitochondrial organization (Fig. 4e and f). However, DNA damage response genes are exclusively enriched in Module 3 (Fig. 4f; Dataset S5), aligning with previous reports implicating Arabidopsis XCT and human PRP19 in DNA repair (Kumimoto et al. 2021; Maréchal et al. 2014). Notably, the role of human PRP19 in DNA damage response is biochemically separable from its splicing activity. Accordingly, our results suggest that DNA damage response genes exhibit distinct regulatory patterns from many RNA metabolic genes in *prp19a-1 prp19b-1* and *xct-2* mutants.

In addition to these commonly regulated modules, we also identified modules that are uniquely affected by either XCT or PRP19. For instance, genes in Modules 5 and 6 are significantly mis-expressed in *xct-2* but not in *prp19a-1 prp19b-1* (Fig. 4b) and are enriched for mRNA metabolism and secondary cell wall biogenesis, respectively (Dataset S5). This suggests that XCT may modulate expression of these genes independently of PRP19. Notably, neither *prp19a-1 xct-2* nor *prp19b-1 xct-2* restores gene expression in these modules, indicating that these genes are unlikely to contribute to the restoration of circadian function. Collectively, our WGCNA results highlight that XCT and PRP19 affect overlapping as well as distinct transcriptional programs, underscoring the complex basis of their pleiotropic phenotypes.

### Expression of anthocyanin biosynthesis-related genes is correlated with circadian phenotypes

**Figure 5:**
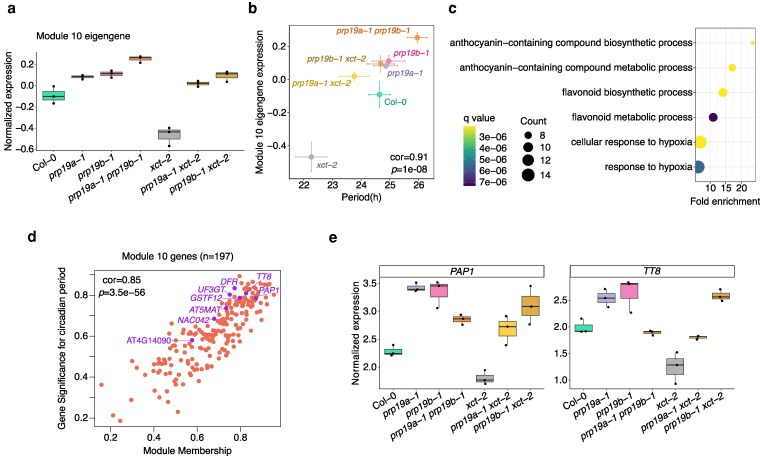
Gene expression in a module enriched for anthocyanin biosynthesis is correlated with circadian clock regulation by *XCT* and *PRP19*. a) Normalized expression of the eigengene for Module 10. Each data point represents a biological replicate from RNA-seq (n = 3). The upper edge, middle line, and lower edge in the boxplot represent the 75% quartile, median, and 25% quartile of the data, respectively. b) Positive Pearson correlation between Module 10 eigengene expression and the circadian period (shown in Fig. 1a) in the indicated genotypes. Horizontal and vertical error bars represent the mean ± SE for circadian period (n = 23 to 36) and eigengene expression (n = 3), respectively. c) Top 6 biological processes, as determined by q values, over-represented in the 197 Module 10 genes relative to all 20,258 expressed genes according to GO analysis. d) Pearson correlation between module membership and gene significance values for the 197 Module 10 genes. Module membership and gene significance correspond to the correlation of a gene's expression profile with module eigengene expression and circadian period length, respectively. Key anthocyanin biosynthesis-related genes are labeled. e) Normalized RNA-seq read counts for the top 2 anthocyanin biosynthesis-related transcription factors highlighted in (d) in the indicated genotypes. Each data point represents a biological replicate from RNA-seq (n = 3). The upper edge, middle line, and lower edge in the boxplot represent the 75% quartile, median, and 25% quartile of the data, respectively.

To uncover molecular mechanisms potentially underlying the antagonistic regulation of circadian period by XCT and PRP19, we analyzed correlations between module eigengenes identified by WGCNA and circadian period lengths across Col-0 and all sequenced *xct* and *prp19* mutants. Among the 21 modules, Modules 5, 6, 9, 10, and 12 exhibit significant positive or negative Pearson correlation with circadian period (*P* < 0.01; Fig. S10a). We focused on Module 10 since it is the only one that meets the following criteria: (i) genes are oppositely regulated in *xct-2* and *prp19a-1 prp19b-1* compared to Col-0; and (ii) gene expression defects are fully restored in both *prp19a-1 xct-2* and *prp19b-1 xct-2* (Fig. 5a; Fig. S10b). Normalized Module 10 eigengene expression shows the highest correlation with circadian period length across all 21 modules (correlation = 0.91; *P* = 1e-08; Fig. 5b; Fig. S10a), establishing a potential link between Module 10 genes and clock regulation mediated by XCT and PRP19. Surprisingly, no known core circadian clock genes are present in this module (Datasets S3 and S4), suggesting that XCT and PRP19 do not affect circadian clock function directly through transcriptional control of core clock genes.

To investigate the biological functions of Module 10 genes, we performed GO enrichment analysis. Genes involved in anthocyanin and flavonoid biosynthesis, as well as cellular responses to hypoxia, are significantly over-represented (q < 1e-5; Fig. 5c; Dataset S5). Anthocyanins are antioxidant pigments that play critical roles in plant responses to various stressors (Li and Ahammed 2023). Several studies have demonstrated connections between the Arabidopsis circadian clock and anthocyanin-related pathways. For instance, expression of many anthocyanin/flavonoid biosynthetic enzymes and transcription factors are circadian regulated (Harmer et al. 2000). Additionally, core clock genes, including *REVEILLE8* (*RVE8*), *LNK1*, *LNK2*, *LIGHT-REGULATED WD 1* (*LWD1*), and *LWD2*, have been previously implicated in anthocyanin production (Wu et al. 2008, 2016; Pérez-García et al. 2015). However, expression of these clock genes in *xct-2* and *prp19a-1 prp19b-1* does not resemble that of the anthocyanin-related genes in Module 10 (Fig. S11), suggesting that XCT and PRP19 control anthocyanin biosynthetic gene expression by a previously unrecognized mechanism.

We next directly examined anthocyanin-related genes within Module 10. Of the 197 genes in this module, 8 are annotated with the GO term “anthocyanin-containing compound metabolic process.” Among these 8 genes, *PRODUCTION OF ANTHOCYANIN PIGMENT 1* (*PAP1*) and *TRANSPARENT TESTA 8* (*TT8*), 2 essential transcriptional regulators of anthocyanin biosynthesis (Falcone Ferreyra et al. 2012), exhibit the highest module membership (Pearson correlation with the Module 10 eigengene) and gene significance (Pearson correlation with circadian period length) (Fig. 5d), and are therefore top candidate genes in Module 10. Additional genes in this group, such as *DIHYDROFLAVONOL 4-REDUCTASE* (*DFR*), *UDP-GLUCOSE:FLAVONOID 3-O-GLUCOSYLTRANSFERASE* (*UF3GT*), *GLUTATHIONE S-TRANSFERASE PHI 12* (*GSTF12*), and *MALONYL-COA:ANTHOCYANIDIN 5-O-GLUCOSIDE-6″-O-MALONYLTRANSFERASE* (*AT5MAT*), encode enzymes involved in flavonoid and anthocyanin biosynthesis (Falcone Ferreyra et al. 2012). Expression of all these genes is significantly reduced in *xct-2* compared to Col-0 and restored in *prp19 xct-2* double mutants (Fig. 5e; Fig. S12), mirroring the circadian period phenotypes in these genotypes. Collectively, these findings indicate that XCT promotes and PRP19 represses the expression of genes encoding key anthocyanin biosynthetic transcription factors and enzymes.

### *XCT* and *PRP19* antagonistically regulate anthocyanin accumulation

**Figure 6:**
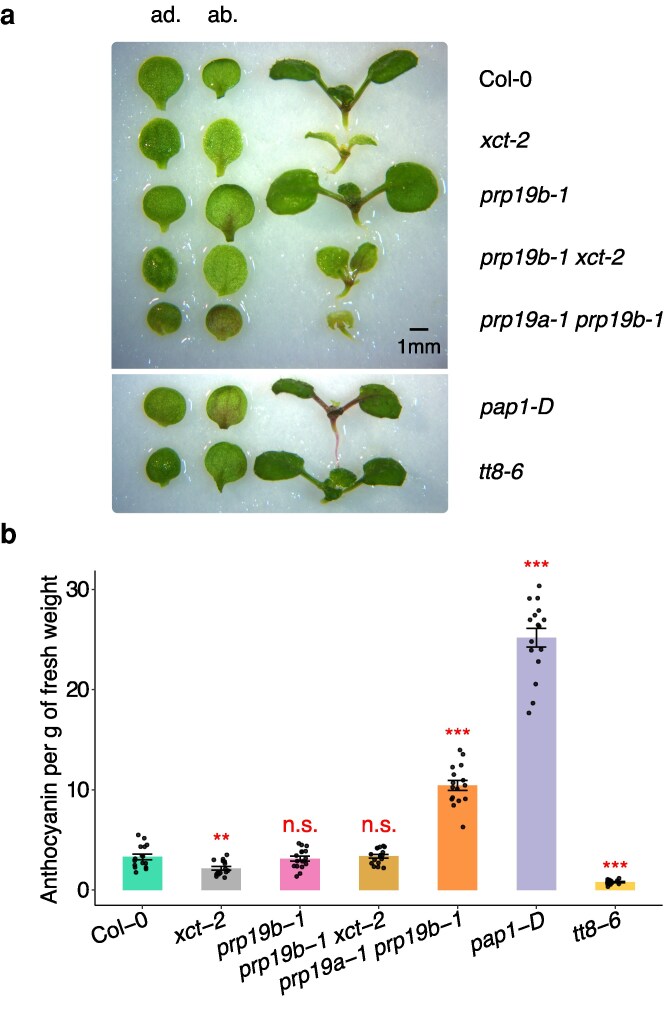
*XCT* and *PRP19* antagonistically regulate anthocyanin accumulation. a) Representative photos showing anthocyanin accumulation in seedlings grown under constant white light for 10 d. Left, adaxial side of a cotyledon (ad.); middle, abaxial side of a cotyledon (ab.); right, a whole seedling without cotyledons. The gain-of-function mutant *pap1-D* and the loss-of-function mutant *tt8-6* serve as positive and negative controls for anthocyanin biosynthesis, respectively. All images are shown at the same scale; the scale bar represents 1 mm. b) Quantification of anthocyanin accumulation. Bar plots represent means ± SE (n = 16). Plants were grown as described in a). Anthocyanin levels were calculated using the formula A530 − 0.25 × A657 and were normalized by fresh weight of the plant tissue. Statistical significance was determined by comparing the indicated genotypes to Col-0 using 2-sided Welch's *t*-test: n.s., not significant; **, *P* < 0.01; ***, *P* < 0.001. Experiments were independently repeated twice with similar results.

To assess whether XCT and PRP19 influence anthocyanin biosynthesis, we measured anthocyanin levels in corresponding mutant lines. For consistency with our RNA-seq and circadian clock assays, we grew all plants under constant light and temperature for 10 d prior to sampling. As positive and negative controls for anthocyanin production, we included *pap1-D* and *tt8-6* mutants, respectively. *pap1-D* is a dominant activation-tagged allele that overexpresses *PAP1* and downstream anthocyanin biosynthetic genes (Borevitz et al. 2000). *tt8-6* is a loss-of-function mutant that results in reduced anthocyanin levels (Rosso et al. 2003). Consistent with previous reports under other light conditions, *pap1-D* displays strong anthocyanin accumulation in the hypocotyl, shoot apex, and leaf vasculature, whereas *tt8-6* shows significantly reduced pigmentation compared to Col-0 (Fig. 6a and b).

We next examined anthocyanin levels in *xct-2*, *prp19b-1*, *prp19b-1 xct-2*, and *prp19a-1 prp19b-1* mutants. Compared to Col-0, *xct-2* seedlings exhibit visibly reduced anthocyanin, particularly near the petiole and shoot apex (Fig. 6a; Fig. S13), as confirmed by quantitative analysis of anthocyanin extracted from whole shoot tissue (Fig. 6b). In contrast, *prp19a-1 prp19b-1* double mutants accumulate more than twice the anthocyanin content of Col-0 (Fig. 6b), with increased pigmentation most visible on the abaxial sides of cotyledons (Fig. 6a). This phenotype is consistent with a recent study showing increased anthocyanin levels in *prp19a-1 prp19b-1* under blue light (Jiang et al. 2023). These data support the opposing roles for XCT and PRP19 in regulating anthocyanin accumulation under constant environmental conditions. Because *prp19b-1 xct-2* double mutants fully restore both the circadian period and anthocyanin gene expression defects observed in *xct-2*, we also examined anthocyanin accumulation in this background. Notably, *prp19b-1 xct-2* seedlings accumulate anthocyanin at levels comparable to the *prp19b-1* single mutant and Col-0 (Fig. 6a and b), indicating that mutation of *PRP19B* fully suppresses the anthocyanin deficiency of *xct-2*. Together, our findings demonstrate that XCT and PRP19 antagonistically regulate both anthocyanin-related gene expression and pigment accumulation. These phenotypes strongly correlate with the effects of XCT and PRP19 on circadian clock function, raising the possibility that mis-regulation of the anthocyanin metabolic pathway might cause changes in circadian clock period.

### Altered anthocyanin accumulation does not affect circadian clock period

**Figure 7:**
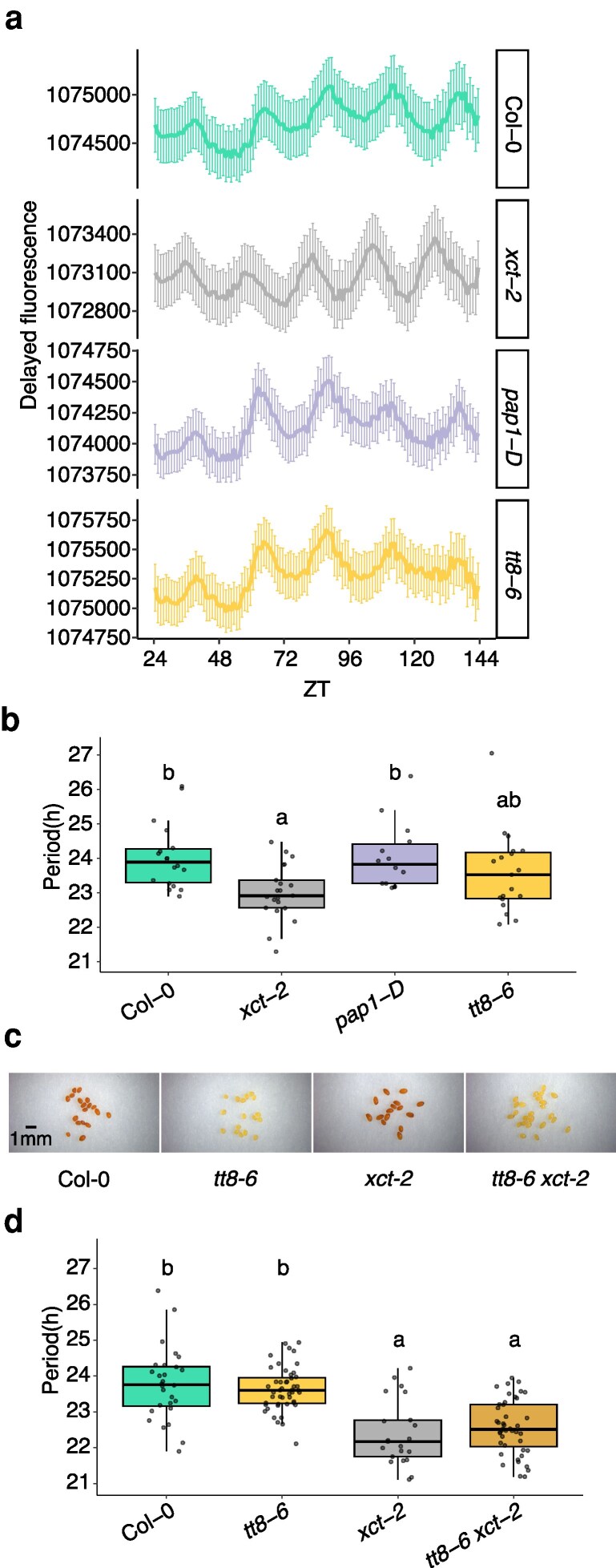
Reduction or induction of anthocyanin accumulation does not alter circadian clock period. a) Delayed fluorescence rhythms in the indicated genotypes. Traces represent mean ± SE (n = 14 to 22) of detrended fluorescence intensity. Fourteen-day-old plants entrained under 12-h white light/12-h dark were transferred at ZT0 to a chamber with constant temperature and light conditions for imaging. b) Circadian period of delayed fluorescence rhythms estimated from a). Statistical significance was determined using a linear regression model with genotype as a fixed effect and is indicated by lower case letters (Tukey's post hoc test for multiple comparisons, *P* < 0.05). c) Severe seed coat pigmentation defects in *tt8-6* and *tt8-6 xct-2* double mutant. d) Circadian period of indicated genotypes as determined using a *CCR2*::*LUC2* reporter. Six-day-old plants were assayed under constant temperature and light conditions. Periods from rhythmic plants with a relative amplitude error (RAE) < 0.8 were plotted (n = 22 to 47). Different letters indicate genotypes with significantly different period estimates as determined by linear regression with genotype as a fixed effect followed by Tukey's post hoc test for multiple comparisons (*P* < 0.05). The upper edge, middle line, and lower edge in the boxplots in b) and d) represent the 75% quartile, median, and 25% quartile of the data, respectively. Experiments were independently repeated 3 times with similar results.

A recent study reported that mutants for genes encoding key enzymes in flavonoid biosynthesis, including *CHALCONE SYNTHASE* (*CHS*) and *FLAVONOID 3′-HYDROXYLASE* (*F3′H*), increase the amplitude of *TOC1* transcriptional rhythms (Hildreth et al. 2022). However, no obvious effect on circadian period length was observed in these mutants. We hypothesized that if XCT and PRP19 regulate circadian period through anthocyanin metabolism, then mutation of key anthocyanin genes mis-regulated in *xct-2* and *prp19a-1 prp19b-1* might alter circadian period. To test this, we measured the circadian period in *pap1-D* and *tt8-6* using a nontransgenic delayed fluorescence assay (Gould et al. 2009). As expected, *xct-2* displays a significantly shorter period of delayed fluorescence rhythms than Col-0 (Fig. 7a and b), consistent with previous analyses of leaf movement and transcriptional rhythms in this allele (Martin-Tryon and Harmer 2008). In contrast, *pap1-D* and *tt8-6* mutants exhibit no significant change in circadian period relative to Col-0 (Fig. 7a and b), despite inducing or reducing anthocyanin levels to a greater extent than observed in *prp19a-1 prp19b-1* and *xct-2*, respectively (Fig. 6b).

To further assess whether the short-period phenotype in *xct-2* is due to reduced anthocyanin biosynthesis, we examined the circadian period of *tt8-6* single and *tt8-6 xct-2* double mutants using the *CCR2*::*LUC2* transcriptional reporter. As expected, *tt8-6* seeds have light yellow seed coats due to severe anthocyanin deficiency, while *xct-2* seeds retain a darker pigmentation (Fig. 7c), reflecting the relatively mild anthocyanin reduction in *xct-2* seedlings (Fig. 6). Consistent with our delayed fluorescence data, *tt8-6* single mutants display a normal period for *CCR2*::*LUC2* rhythms relative to Col-0 (Fig. 7d). *tt8-6 xct-2* double mutants also produce yellow seeds, indicating that *tt8-6* further reduces anthocyanin biosynthesis in the *xct-2* background (Fig. 7c). Despite this enhancement of anthocyanin deficiency, *tt8-6 xct-2* double mutants show no additional shortening of circadian period compared to *xct-2* alone (Fig. 7d). Together, these results indicate that XCT does not regulate circadian period directly by its effects on anthocyanin biosynthesis.

### *CBF* family genes do not mediate circadian clock period regulation

We next investigated whether other Module 10 genes with known connections to circadian clock function might mediate XCT's regulation on circadian clock period. Notably, expression of 4 of the 6 total *C-REPEAT-BINDING FACTOR* (*CBF*) family genes, including *CBF1*, *CBF2*, *CBF4*, and *DWARF AND DELAYED FLOWERING 1* (*DDF1*), strongly correlates with both circadian period length and the Module 10 eigengene (Fig. S14a). Expression of these genes is downregulated in *xct-2* and fully restored in *prp19 xct-2* double mutants (Fig. S14b), suggesting that XCT regulates *CBF* expression in a PRP19-dependent manner. *CBF* genes are well characterized for their roles in cold and drought stress responses (Nakashima et al. 2025). Previous studies have established that low-temperature induction of *CBF1*, *CBF2*, and *CBF3* expression is controlled by the circadian clock (Dong et al. 2011; Fowler et al. 2005). Moreover, CBF1 has been shown to integrate cold signals into the clock by directly regulating expression of a core circadian clock gene (Chow et al. 2014). To test whether *CBF* genes contribute to the short circadian period in *xct-2*, we examined delayed fluorescence rhythms in plants mutant for *cbf1 cbf2 cbf3* (Xie et al. 2021). These triple mutant plants exhibit no significant difference in circadian period length compared to Col-0 (Fig. S14c and d). Thus, the shortened circadian period in *xct-2* is likely not mediated by loss of function of *CBF* genes.

## Discussion

Precise control of biological timing in plants relies on interconnected transcriptional–translational feedback loops, as well as post-transcriptional and post-translational mechanisms (Harmer 2025). We previously showed that *XCT* and the 2 functionally redundant homologs of human *PRP19*, *PRP19A* and *PRP19B*, act antagonistically to regulate Arabidopsis circadian period length within a shared genetic pathway (Zhang et al. 2023). XCT and PRP19 proteins physically interact with each other and with multiple spliceosome-associated proteins, contributing to genome-wide pre-mRNA splicing (Jia et al. 2017; Li et al. 2019; Liu et al. 2022; Zhang et al. 2023). These findings raised the possibility that XCT and PRP19 influence circadian rhythms by modulating splicing. However, here we report that the *xct-2* single mutant and the *prp19a-1 prp19b-1* double mutant exhibit distinct splicing defects (Fig. 1b and d).

Notably, although the short-period phenotype of *xct-2* is suppressed in both *prp19a-1 xct-2* and *prp19b-1 xct-2* double mutants (Fig. 1a), the number of differentially spliced genes and the overall severity of splicing defects remain unchanged among these genotypes (Fig. 1c to f). Together, these results suggest that the opposing effects of XCT and PRP19 on circadian period are likely mediated through mechanisms that are independent of their roles in splicing.

Pre-mRNA splicing is typically coupled to the transcription machinery and occurs co-transcriptionally (Moore and Proudfoot 2009). Consistent with this, spliceosome-associated proteins often play additional roles in transcriptional regulation (Tognacca et al. 2020). Many of these proteins are core components or associated factors of the PRP19 complex. For instance, SPLICEOSOMAL TIMEKEEPER LOCUS1 (STIPL1), the Arabidopsis homolog of the spliceosome disassembly factor NTC-RELATED PROTEIN 1 (NTR1) in yeast and humans, co-localizes with RNA Pol II and influences transcriptional elongation of protein- and miRNA-coding genes (Dolata et al. 2015; Wang et al. 2019). Similarly, CELL DIVISION CYCLE 5 (CDC5), a core component of the PRP19 complex, also acts as a putative MYB-related transcription factor to enhance pri-miRNA biogenesis by recruiting RNA Pol II to miRNA gene promoters (Hirayama and Shinozaki 1996; Zhang et al. 2013), and to delay flowering by transcriptionally activating *FLOWERING LOCUS C* (*FLC*) expression (Xin et al. 2024). An additional example is SKI-INTERACTING PROTEIN (SKIP), another core member of the PRP19 complex. SKIP interacts with distinct protein complexes to independently regulate mRNA splicing and transcriptional activation of flowering genes (Li et al. 2019). Notably, loss-of-function mutants of *STIPL1*, *CDC5*, and *SKIP* all display a lengthened circadian period relative to Col-0, resembling the phenotype of *prp19a prp19b* double mutants (Jones et al. 2012; Wang et al. 2012; Zhang et al. 2023). These findings raise the possibility that components of the PRP19 complex may broadly influence circadian clock pace through splicing-independent transcriptional functions.

Given that several PRP19 complex-associated splicing factors influence transcription, it is plausible that XCT and PRP19 also contribute to transcriptional regulation through splicing-independent mechanisms. Supporting this idea, a previous transcriptome analysis found no correlation between splicing defects and gene expression in *prp19a-1 prp19b-1* mutants, suggesting that PRP19 may regulate gene expression independently of its splicing role (Jia et al. 2017). Supporting direct roles for PRP19 proteins in transcription, PRP19A was recently identified as a chromatin-binding protein (Jiang et al. 2023). While direct transcriptional regulatory activity of XCT has not yet been demonstrated in Arabidopsis, chromatin association has been reported for XCT homologs in fission yeast, green algae, and mice (Anver et al. 2014; Li et al. 2018a; Wang et al. 2025). In addition, a recent proteomic study found that XCT co-purifies with several transcription-related proteins, including RNA Pol II subunits and members of the PAF1 complex, which is known to mediate transcriptional elongation and histone modification (Gao et al. 2024). Given the conserved sequence and splicing functions of XCT and PRP19 homologs across eukaryotes, their chromatin-binding and transcriptional regulatory roles may likewise be evolutionarily conserved.

To investigate a possible role for XCT and PRP19 in transcriptional regulation, we examined their genome-wide impact on gene expression. Our RNA-seq data reveal a significant overlap in DEGs between *xct-2* and *prp19a-1 prp19b-1* mutants (Fig. 3). WGCNA analyses further suggest that many of these genes are likely co-regulated by both factors (Fig. 4). Interestingly, we found that expression of some genes mis-regulated in *xct-2* is altered in the opposite direction in *prp19a-1 prp19b-1*, and that these changes are restored to near wild-type levels in *prp19a-1 xct-2* and *prp19b-1 xct-2* double mutants (Fig. 5a and b; Fig. S8b). Although we cannot fully rule out the possibilities that these gene expression changes are caused by low-abundance splicing events that are undetectable at the RNA-seq read depth in our experiment, or by secondary effects of splicing (eg, mis-splicing of transcription factors), several lines of evidence suggest that these explanations are unlikely. First, despite sharing many DEGs, *xct-2* and *prp19a-1 prp19b-1* mutants show minimal overlap in differentially spliced genes (Figs. 1b and 2a). Second, genes showing the strongest fold changes of expression in *xct-2* are not subject to differential splicing (Fig. 2b). Third, we observe a significant increase in transposable element expression in *xct-2* (Fig. S7), consistent with previous findings in the fission yeast Δ*xap5* mutant (Anver et al. 2014) and suggesting that XCT homologs may play a conserved role in transposon repression. Together, these results suggest that XCT and PRP19 influence gene expression through mechanisms that are independent of splicing, similar to the functions of other PRP19 complex-associated proteins, such as STIPL1, CDC5, and SKIP.

Beyond circadian regulation and pre-mRNA splicing, XCT and PRP19 have been implicated in a variety of shared biological processes, including small RNA biogenesis, immune responses, and photomorphogenesis (Martin-Tryon and Harmer 2008; Monaghan et al. 2009; Ellison et al. 2011; Fang et al. 2015; Jia et al. 2017; Xu et al. 2017; Li et al. 2018b; Kumimoto et al. 2021; Jiang et al. 2023). Although similar phenotypes have been reported separately in either *xct-2* or *prp19a-1 prp19b-1* mutants, any shared molecular basis for their pleiotropic phenotypes remains poorly understood. Our transcriptome profiling suggests that co-regulation of gene expression may contribute to some of these shared phenotypes. For example, genes broadly involved in RNA metabolism are co-upregulated in *xct-2* and *prp19a-1 prp19b-1* mutants (Figs. 3 and 4; Dataset S5). This is consistent with reports of reduced splicing efficiency and impaired miRNA and small interfering RNA production in these genotypes (Fang et al. 2015; Jia et al. 2017; Li et al. 2018b; Liu et al. 2022; Zhang et al. 2023). In addition, we observed a coordinated downregulation of photosynthesis- and root development-related genes, along with upregulation of DNA damage response genes in *xct-2*, *prp19a-1 prp19b-1*, and *prp19 xct-2* double mutants (Fig. 4). Notably, while XCT or PRP19 have been individually implicated in these pathways (Martin-Tryon and Harmer 2008; Kumimoto et al. 2021; Gao et al. 2024; Yu et al. 2024), their shared involvement has not been previously elucidated. These findings raise the possibility that XCT and PRP19 act in concert to transcriptionally modulate multiple physiological processes. Further dissection of their molecular functions will help clarify how these pleiotropic regulators contribute to the shared regulation of diverse cellular and developmental pathways.

While most DEGs in *xct-2* and *prp19a-1 prp19b-1* mutants are co-upregulated or co-downregulated relative to Col-0, a subset of genes is oppositely regulated between the 2 genotypes (Fig. 4b; Fig. S8). We speculate that these oppositely regulated genes may contribute to processes that are antagonistically modulated by XCT and PRP19, such as circadian period length. Here, we report that anthocyanin biosynthesis is another physiological process under antagonistic regulation by XCT and PRP19 (Figs. 5 and 6). Specifically, XCT promotes, while PRP19 suppresses, anthocyanin biosynthetic gene expression and pigment accumulation under constant light and temperature conditions (Figs. 5 and 6). Strikingly, anthocyanin levels in the *prp19b-1 xct-2* double mutant are restored to wild-type and *prp19b-1* single mutant levels (Fig. 6), indicating that the positive effect of XCT on anthocyanin accumulation depends on functional PRP19B.

Interestingly, while *xct-2* mutants exhibit reduced expression of key anthocyanin biosynthetic genes, such as *PAP1*, *TT8*, and *DFR*, genes associated with other branches of the flavonoid pathway, such as *FLAVONOL SYNTHASE 1* (*FLS1*), *MYB DOMAIN PROTEIN 11* (*MYB11*), *MYB12*, and *MYB111*, are upregulated (Fig. S6a; Dataset S2). It has been reported that perturbation in 1 branch of flavonoid biosynthesis pathways can affect the others (Yu et al. 2023). For instance, the *myb11 myb12 myb111* triple mutant displays reduced flavanol accumulation but a slight increase in anthocyanin levels (Stracke et al. 2007). Based on these findings, we propose 2 possible mechanisms for the reduced anthocyanin levels observed in *xct-2*: (i) XCT may activate transcription of anthocyanin biosynthetic genes, and/or (ii) XCT may suppress competing flavonoid pathways, and loss of this suppression in *xct-2* may indirectly inhibit anthocyanin production via metabolic feedback.

### The circadian clock is another process antagonistically regulated by XCT and PRP19

Given the strong correlation between anthocyanin biosynthetic gene expression and circadian period length, we hypothesized that anthocyanin levels might influence circadian clock pace. However, our data do not support this hypothesis. Reducing anthocyanin accumulation via the *tt8-6* mutation in either Col-0 or *xct-2* background does not cause a period-shortening phenotype as observed in *xct-2* (Fig. 7). Likewise, overaccumulation of anthocyanin in the *pap1-D* mutant does not result in a lengthened circadian period (Fig. 7a and b). These findings are consistent with a previous study reporting that *tt4/chs* and *tt7/f3′h* mutants, which disrupt early steps in flavonoid biosynthesis, increase the amplitude of *TOC1* expression rhythms but do not alter period length (Hildreth et al. 2022). Together, these results indicate that anthocyanin levels themselves do not influence the pace of the circadian clock. Instead, the strong correlation between anthocyanin biosynthesis and circadian period length likely reflects co-regulation by an upstream process that involves both XCT and PRP19.

The mechanisms by which XCT and PRP19 antagonistically influence the circadian clock, anthocyanin biosynthesis, and expression of other stress-responsive genes, such as the *CBFs*, remain unclear. Previous studies have shown that core clock components *RVE8*, *LNK1*, and *LNK2* antagonistically regulate anthocyanin biosynthesis by directly binding to the promoters of key anthocyanin-related genes, including *TRANSPARENT TESTA 18 (TT18)* and *CHS* (Pérez-García et al. 2015). Although our RNA-seq data show no opposite regulation of transcript levels or splice isoforms of *RVE8*, *LNK1*, or *LNK2* in *xct-2* and *prp19a-1 prp19b-1* (Fig. S11; Fig. 1f), it remains possible that XCT and PRP19 affect the activity of these or other clock regulators at the protein level, such as has recently been shown for the key clock protein ELF3 (Kumimoto et al. 2026).

Another possibility is that XCT and PRP19 directly affect signaling pathways that are tightly integrated with both circadian regulation and anthocyanin production. One such pathway is light signaling: High light intensity can both induce anthocyanin accumulation and shorten circadian period length (Somers et al. 1998; Maier and Hoecker 2015). Several key regulators of light signaling in Arabidopsis, including *PHYTOCHROME A* (*PHYA*), *PHYB*, *CONSTITUTIVE PHOTOMORPHOGENIC1* (*COP1*), *ELONGATED HYPOCOTYL 5* (*HY5*), *PHYTOCHROME INTERACTING FACTOR 3* (*PIF3*), and *PIF4*, are known to play dual roles in modulating anthocyanin biosynthesis and circadian clock function (Kunkel et al. 1996; Shin et al. 2007, 2013; Maier et al. 2013; Liu et al. 2021). Consistent with this, both *xct-2* and *prp19a-1 prp19b-1* mutants display altered light-responsive hypocotyl phenotypes (Jiang et al. 2023; Martin-Tryon and Harmer 2008), suggesting that XCT and PRP19 might act through light signaling pathways to co-regulate clock function and anthocyanin biosynthesis. Future investigation into the transcriptional and post-transcriptional roles of XCT and PRP19 will shed light on how plants integrate environmental signals with metabolic and circadian regulation.

## Materials and methods

### Plant materials and growth conditions

All Arabidopsis (*Arabidopsis thaliana*) plants used in this study were of the Columbia-0 (Col-0) ecotype. The *xct-2* (SALK_108639), *prp19a-1* (SALK_089300), *prp19b-1* (SALK_050811), *pap1-D* (CS3884), *tt8-6* (GK-241D05), and *cbf1/2/3* (CS73345) mutants have been previously described (Borevitz et al. 2000; Alonso et al. 2003; Rosso et al. 2003; Martin-Tryon and Harmer 2008; Monaghan et al. 2009; Xie et al. 2021). All double mutants used in this study were generated by crossing. Col-0 plants carrying a *CCR2::LUC+* (Strayer et al. 2000) or *CCR2::LUC2* (Hughes and Harmer 2023) transgene were crossed into corresponding mutant backgrounds to introduce luciferase reporters. Genotyping primers used in this study are described in Dataset S6.

For RNA sequencing and anthocyanin content analysis, Arabidopsis seeds were surface sterilized with 70% ethanol containing 0.1% (v/v) Triton X-100 (Sigma) for 5 min, followed by treatment with 100% ethanol for 20 min. For circadian clock period assays, seeds were surface sterilized by chlorine gas in a sealed desiccator for 3 h (50 mL 100% bleach + 3 mL concentrated HCl). After sterilization, seeds were plated on 1× Murashige and Skoog (MS) medium (pH 5.7) containing 0.7% agar and stratified at 4 °C in darkness for 3 d. Germination was carried out at 22 °C under continuous white light (55 μmol m⁻^2^ s⁻^1^) or 12-h light/12-h dark cycles, depending on the experimental design.

### RNA sequencing sample preparation

To minimize the impact of circadian period length variation on differential gene expression analysis, seedlings were germinated and grown in constant light and temperature for 10 d, and samples were then collected at 2-h intervals across a circadian cycle (22 to 26 h, depending on genotype) and pooled equally (Fig. S1). The period length for each tested genotype is shown in Fig. 1a. Approximately 65 mg of pooled seedling tissue was collected for each genotype and each of the 3 biological replicates.

Samples were flash frozen in liquid nitrogen and ground into fine powder using a bead beater. RNA was extracted using Trizol reagent (Invitrogen) following the manufacturer's instructions. Total RNA was treated with DNase I and purified using the RNA Clean & Concentrator Kit (Zymo Research). RNA quantification and quality control were performed using a Nanodrop spectrophotometer and a LabChip GX system. Poly(A)-enriched mRNA libraries were prepared using the KAPA Hyper mRNA library kit. A total of 21 multiplexed libraries were sequenced at the UC Davis DNA Technologies & Expression Analysis Core (https://dnatech.ucdavis.edu/element-biosciences-aviti-sequencing) using paired-end 150 (PE150) mode on an Element Biosciences AVITI sequencer.

### Raw RNA-seq data processing

Quality control of raw sequencing reads was performed using FastQC (v0.11.9) (https://www.bioinformatics.babraham.ac.uk/projects/fastqc/) and MultiQC (v1.15) (Ewels et al. 2016). Adapter trimming, quality filtering (≥20), and read length filtering (≥36 bp) were conducted using Trimmomatic (v0.39) (Bolger et al. 2014). The trimmed reads were then mapped to the *Arabidopsis thaliana* genome (TAIR10 genome assembly, Araport11 annotation) (Cheng et al. 2017) using HISAT2 (v2.2.1) (Kim et al. 2019), with the parameter --max-intronlen 12000 to accommodate *Arabidopsis* intron sizes. Transcript assembly was performed using StringTie (v2.2.1) (Pertea et al. 2016). Gene expression was quantified by counting the number of paired-end reads mapped to unique genomic loci using featureCounts (v2.0.6) (Liao et al. 2014). A summary of processed read counts at each step is provided in Table S1.

### Differential splicing analysis

Differential splicing analysis was performed using the ASpli R package (v2.14.0) (Mancini et al. 2021). The Araport11 genome annotation was first converted into a TxDb object using the GenomicFeatures R package (v1.56.0) (Lawrence et al. 2013). Subgenic features, including exon and intron bins and splice junctions, were extracted using the binGenome function. Read counting for genes and subgenic features was performed with a maximum expected intron size of 12,000 bp and a minimum read length of 36 bp.

Percent splicing index (PSI) and percent intron retention (PIR) were used to quantify inclusion evidence for each subgenic feature. Exon and intron bins and junctions were included in differential analysis if they (i) had at least 5 supporting reads in 1 or more genotypes and (ii) belonged to genes with an average of at least 10 reads per genotype. Differential splicing events were identified using bin signals [inclusion > 0.2, false discovery rate (FDR) < 0.05], anchorage signals (inclusion > 0.3, FDR < 0.01), and locale signals (inclusion > 0.3, FDR < 0.01). Splicing events were classified using the Araport11 annotation and custom scripts.

### Differential gene expression analysis

Differential gene expression analysis was conducted using the edgeR R package (v4.2.2) (Chen et al. 2025). Genes with low expression (<10 counts in at least 3 libraries) were removed, leaving 20,258 genes for analysis (Dataset S3). Read counts were normalized using the trimmed mean of M-values (TMM) method. Differential expression was determined by quasi-likelihood (QL) F-tests using the glmQLFit function in edgeR. Genes were considered differentially expressed relative to wild-type Col-0 if they had a fold change > 1.2 and an adjusted *P* < 0.05 (Benjamini and Hochberg correction). PCA was performed on log_2_-transformed TMM-normalized expression data. Genes encoding transposable elements were identified using Araport11 annotation.

### Weighted gene co-expression network analysis

WGCNA was performed using the WGCNA R package (v1.72-5) (Langfelder and Horvath 2008). Genes with low expression levels (<10 counts in at least 3 libraries) were removed. To assess expression variability across genotypes, the coefficient of variation of log_2_-transformed TMM-normalized expression values was calculated. The least variable 25% of genes were filtered out, leaving 15,193 genes for co-expression module detection.

An adjacency matrix was constructed using a soft-thresholding power of 10 and transformed into a topological overlap matrix (TOM). To construct a signed network, type (for adjacency calculation) and TOMType (for topological overlap calculation) were set to “signed hybrid” and “signed Nowick,” respectively. A dissimilarity matrix was computed from TOM, and hierarchical clustering was performed using the unweighted average linkage method. Gene modules were identified using the dynamic tree cut method, with a minimum module size of 30. Modules with similar expression profiles were merged using a height cut of 0.2, resulting in 21 co-repressed gene modules. A module eigengene was defined as the first PC of the expression matrix for each module. Module membership and gene significance were calculated as the Pearson correlation between individual gene expression and the module eigengene, and between individual gene expression and circadian period length, respectively.

### GO analysis

GO enrichment analysis was performed using the R package clusterProfiler (v4.10.1) (Yu et al. 2012), with GO annotations retrieved from org.At.tair.db (v3.18.0) (Carlson 2017). Enriched GO terms were identified using all 20,258 expressed genes as the background, with an FDR < 0.05 threshold. Redundant GO terms were removed using the simplify function in clusterProfiler with a similarity cutoff of 0.75 for *P* value.

### Luciferase and delayed fluorescence assays for circadian clock period analysis

For plants carrying the *CCR2::LUC+* reporter, approximately 10 to 15 seeds were pipetted as a clump onto 1× MS medium. For plants expressing the brighter *CCR2::LUC2* reporter, individual seeds were placed separately. A sterile straw fragment was positioned around the seeds to enhance signal detection as previously described (Zhang and Harmer 2024). Seedlings were entrained in 12-h light (55 μmol m⁻^2^ s⁻^1^)/12-h dark cycles at 22 °C for 6 d and then sprayed with 3 mM D-luciferin (Biosynth) in 0.01% (v/v) Triton X-100. For delayed fluorescence assays, clusters of 15 to 20 seeds were grown under the same light/dark cycles at 22 °C for 14 d until true leaves were fully developed.

Plants were then transferred to constant conditions (22 °C and continuous red and blue light at 35 μmol m⁻^2^ s⁻^1^ each, provided by the XtremeLUX LED Lighting System) for imaging. Bioluminescence and fluorescence were imaged using a cooled CCD camera [ORCA-R2 {Hamamatsu}] with exposure times of 20, 1, and 2 min for *CCR2::LUC+*, *CCR2::LUC2*, and delayed fluorescence assays, respectively. Signal intensity was quantified using the “Graph Intensities” function in MetaMorph 7.7.1.0 (Molecular Devices). Circadian period and rhythmicity were analyzed using BioDare2 (https://biodare2.ed.ac.uk) (Zieliński et al. 2022). Raw data were linearly detrended and fit to a cosine wave using FFT-NLLS (Plautz et al. 1997). Samples with a relative amplitude error (ERR) < 0.6 were considered rhythmic and included in period analysis, unless stated otherwise.

### Measurement of anthocyanin content

Anthocyanin content was measured as previously described with minor modifications (Mita et al. 1997). Five to eight 10-d-old seedlings grown under continuous white light (55 μmol m⁻^2^ s⁻^1^) at 22 °C were pooled per sample. Roots were removed to ensure only above-ground tissue was analyzed. Samples were weighed and incubated in 900 μL freshly prepared extraction buffer [1% {v/v} HCl in methanol] at 4 °C in the dark with gentle shaking for 24 h. Absorbance of the supernatant was measured at 535 and 650 nm. Relative anthocyanin content was calculated as (A530 − 0.25 × A657) per gram of fresh weight.

### Accession numbers

All the *A. thaliana* genes studied in this paper can be found under the following accession numbers:

*XCT*, AT2G21150

*PRP19A*, AT1G04510

*PRP19B*, AT2G33340

*LHY*, AT1G01060

*LNK2*, AT3G54500

*TOC1*, AT5G61380

*TIC*, AT3G22380

*PRR7*, AT5G02810

*PAP1*, AT1G56650

*TT8*, AT4G09820

*TTG1*, AT5G24520

*LWD1*, AT1G12910

*LWD2*, AT3G26640

*RVE8*, AT3G09600

*LNK1*, AT5G64170

*AT5MAT*, AT3G29590

*DFR*, AT5G42800

*GSTF12*, AT5G17220

*UF3GT*, AT5G54060

*CBF1*, AT4G25490

*CBF2*, AT4G25470

*CBF4*, AT5G51990

*DDF1*, AT1G12610

## Supplementary Material

kiag235_Supplementary_Data

## Data Availability

The RNA-seq data are available in NCBI Sequence Read Archive (SRA; accession number: PRJNA1136024). The RNA-seq data analysis scripts are available at GitHub (https://github.com/hzhang-plb/AVITI_2023.git).
