# Supplementary material for: XAP5 CIRCADIAN TIMEKEEPER coordinates circadian rhythms and anthocyanin biosynthesis independently of splicing
Source: Plant Physiol. 2026 Apr 22;201(2):kiag235. doi: 10.1093/plphys/kiag235 (PMC13227948; doi:10.1093/plphys/kiag235)
Supplement: kiag235_Supplementary_Data [file kiag235_supplementary_data.zip › Supplementary Figures Revised.pdf]

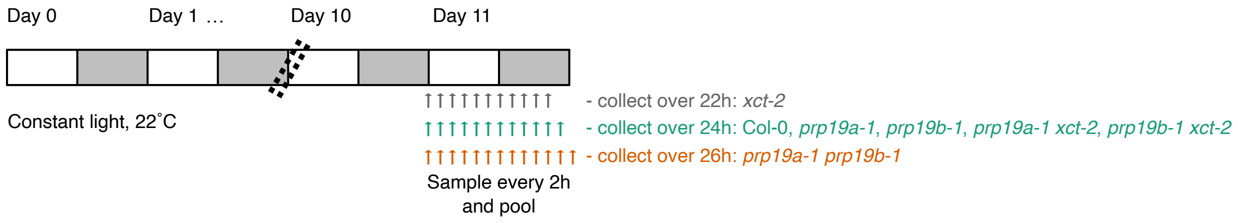

Supplementary Figure S1. Experimental design for RNA-seq experiment. Schematic of sample collection for RNA-seq experiment. White and gray boxes represent subjective day and night, respectively, under constant light and temperature. Arrows indicate time points of sample collection.

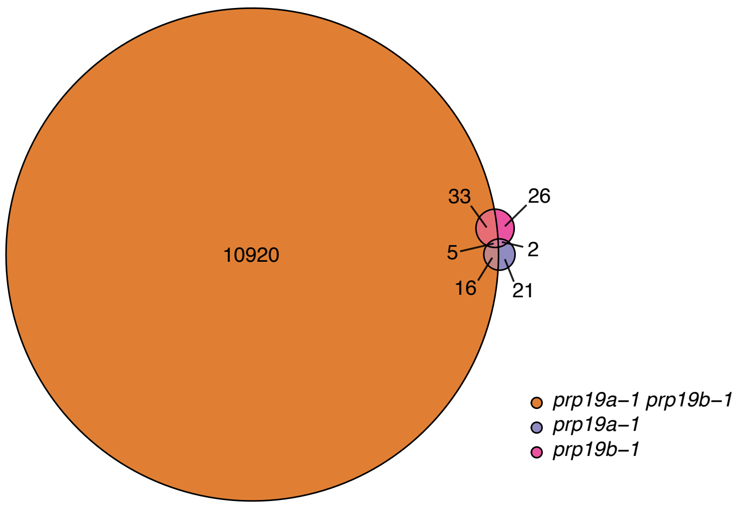

Supplementary Figure S2. PRP19A and PRP19B function redundantly to promote mRNA splicing. Very few differential splicing events are detected in *prp19a-1* and *prp19b-1* single mutants compared to those found in the *prp19a-1 prp19b-1* double mutant relative to Col-0.

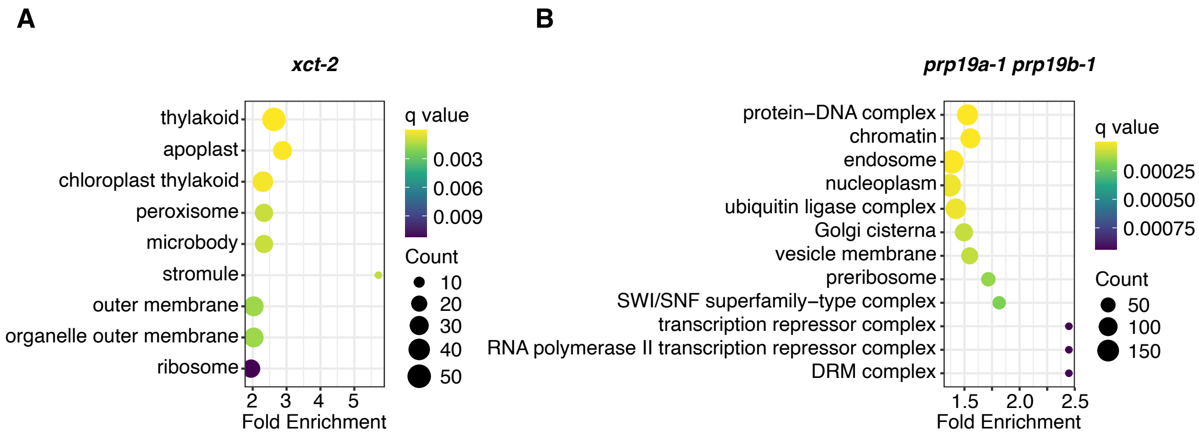

Supplementary Figure S3. Differentially spliced genes in *xct-2* and *prp19a-1 prp19b-1* are enriched for distinct Gene Ontology terms. A) - B) Cellular Components that are over-represented among the 806 and 6,834 genes that contain at least one differential splicing event in *xct-2* A) and *prp19a-1 prp19b-1* B), respectively. All enrichments are determined by Gene Ontology analysis and are relative to the 20,258 expressed genes.

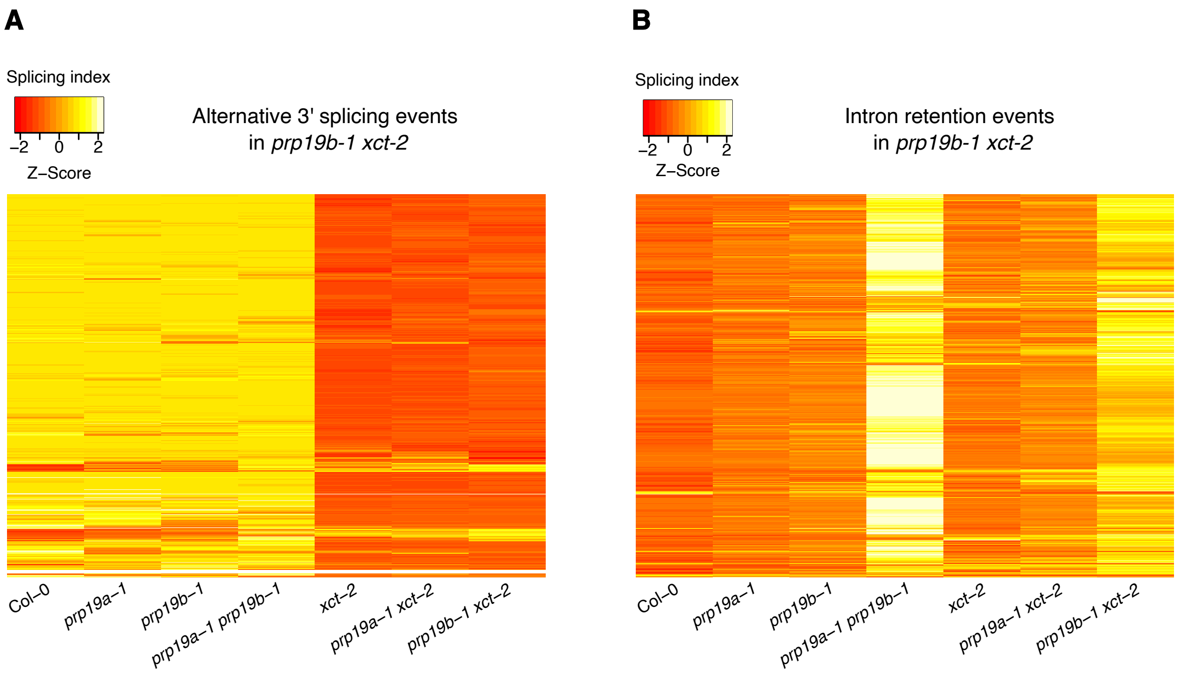

Supplementary Figure S4. ***prp19b-1 xct-2* exhibits similar differential 3' splice site events as *xct-2* and similar intron retention events as *prp19a-1 prp19b-1.*** A) Z-score transformed splicing indices for all 3′ splice site events significantly different in *prp19b-1 xct-2* relative to Col-0 are plotted for the indicated genotypes. Each row represents one event. Lower splicing index values indicate higher usage of alternative 3' splice sites. B) Z-score transformed splicing indices for all intron retention events significantly different in *prp19b-1 xct-2* relative to Col-0 plotted for the indicated genotypes. Higher splicing index values reflect greater intron retention.

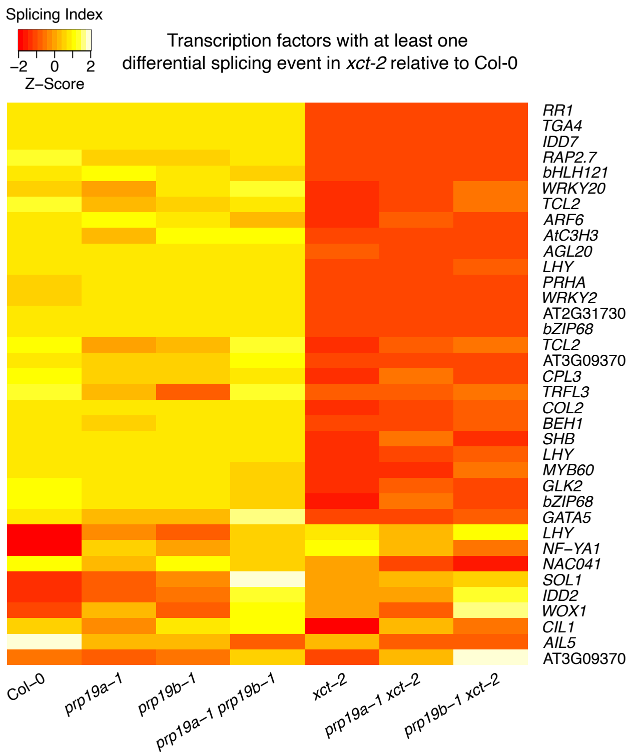

Supplementary Figure S5. Differential splicing of transcription factors in *xct-2* is not restored in *prp19 xct-2* double mutants. Z-score transformed splicing indices for all Arabidopsis transcription factors (Jin et al., 2017) with differential splicing in *xct-2* relative to Col-0 are plotted for the indicated genotypes. Each row represents a unique splicing event in the indicated transcription factor. Lower splicing index values indicate higher usage of alternative 3' splice sites or a lower degree of intron retention.

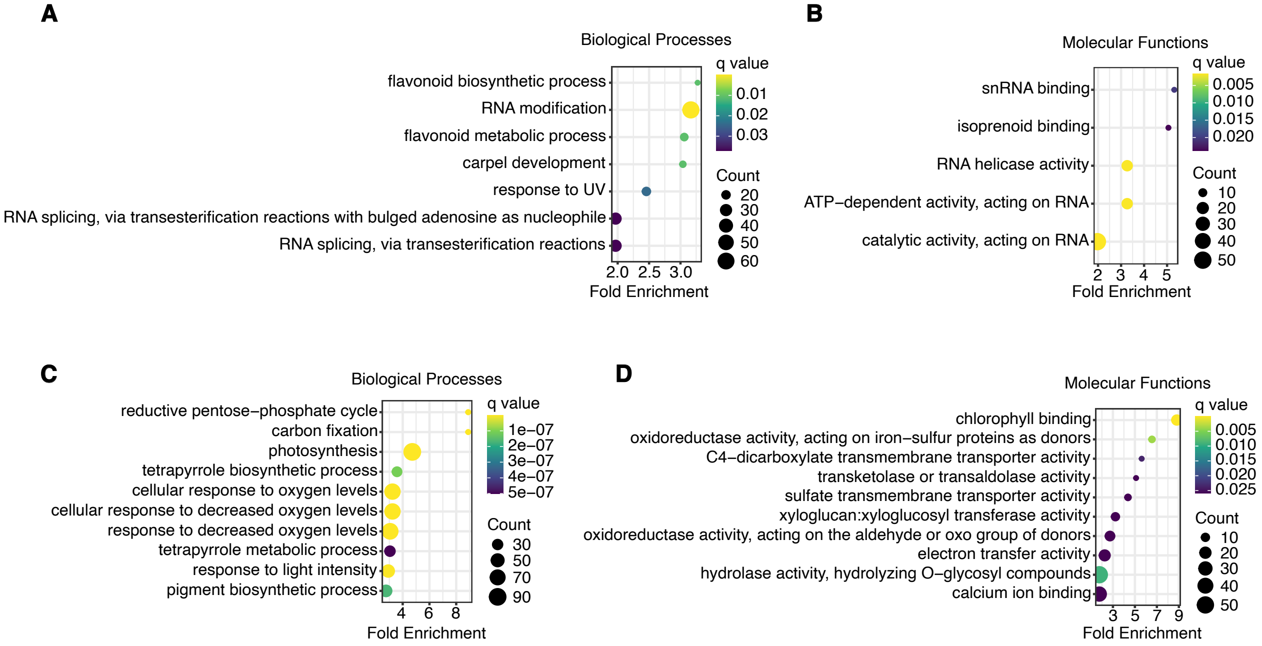

Supplementary Figure S6. Differentially expressed genes in *xct-2* are enriched for splicing- and photosynthesis-related processes. A) - B) Biological processes and molecular functions that are over-represented among the 1,469 genes upregulated in *xct-2*. C) - D) Biological processes and molecular functions that are over-represented among the 2,099 genes downregulated in *xct-2*. All enrichments are determined by Gene Ontology analysis and are relative to the 20,258 expressed genes.

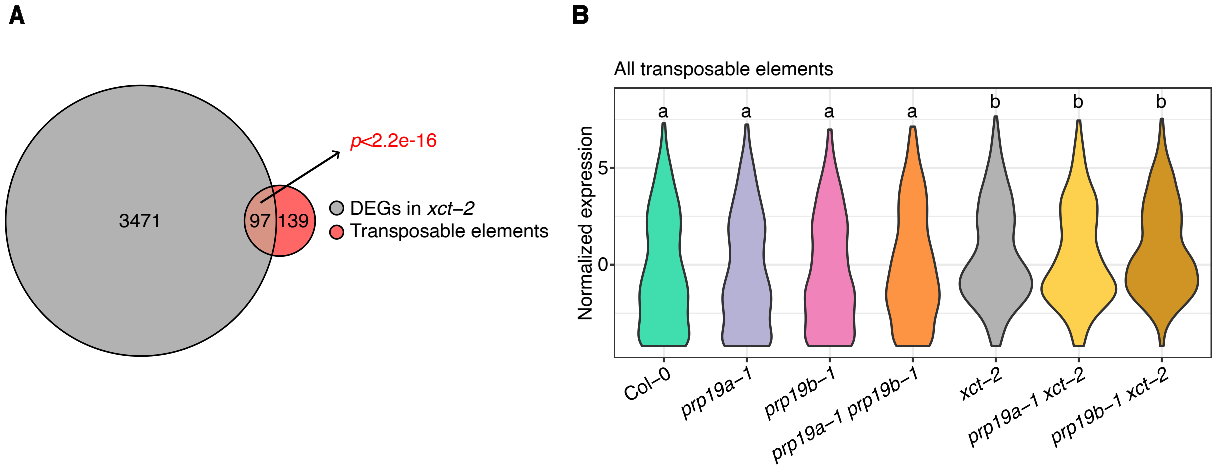

Supplementary Figure S7. Transposable elements are upregulated in both *xct-2* and *prp19 xct-2* double mutants. A) Significant overlap between the 3,568 DEGs in *xct-2* and the 236 transposable elements annotated in the Araport11 genome annotation. Statistical significance of overlapping genes was determined using one-tailed Fisher's exact test. B) Violin plots of normalized RNA-seq read counts of all 236 transposable elements in the indicated genotypes. Different letters indicate genotypes with significantly different transposable element expression as determined by linear regression with genotype as a fixed effect followed by Tukey's post hoc test for multiple comparisons ($p < 0.05$).

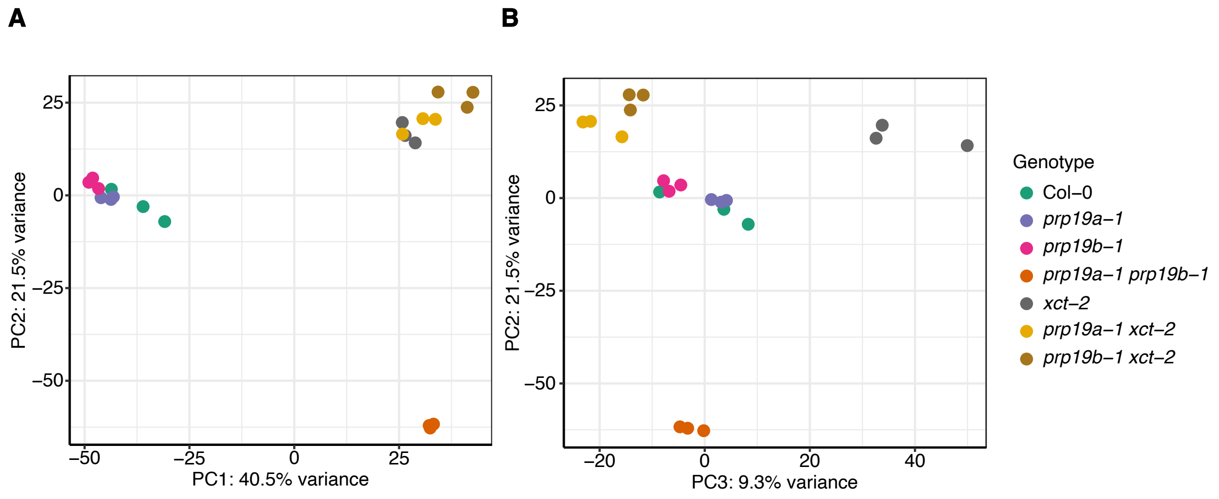

Supplementary Figure S8. Principal component analysis indicates partial restoration of gene expression in *prp19 xct-2* double mutants. A) The first two principal components (PC1 and PC2) separate *xct-2* and *prp19a-1 prp19b-1* double mutants from Col-0 and from each other. B) PC3 separates *prp19 xct-2* double mutants from *xct-2* single mutant. Values on the axes represent the scores for each genotype on the corresponding PC. Each data point represents an independent biological replicate in the RNA-seq experiment. Percent of variance in gene expression explained by each PC is labelled by the axes.

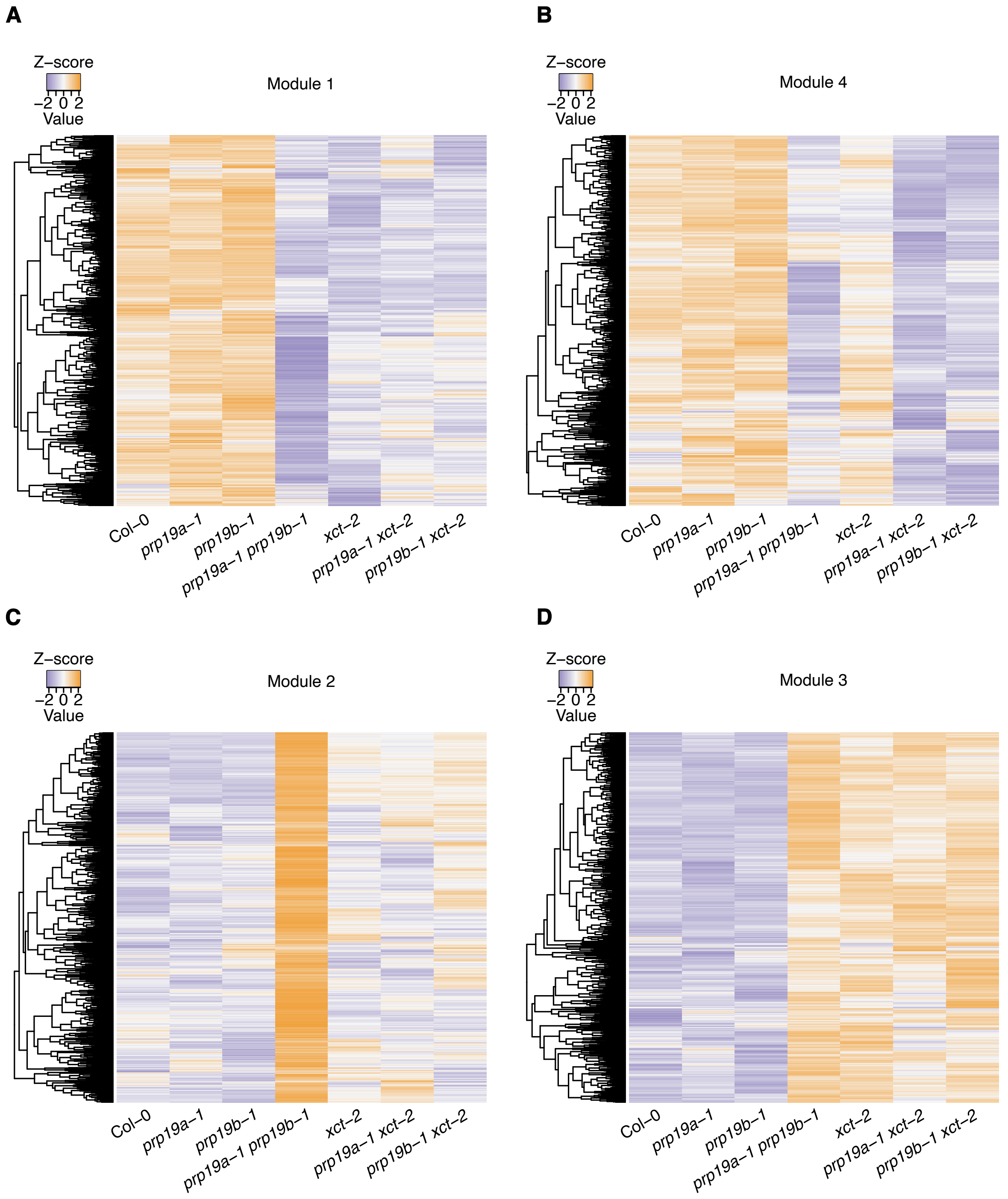

Supplementary Figure S9. WGCNA identifies modules representing distinct expression patterns of genes co-regulated by *XCT* and *PRP19*. A) - D) Z-score transformed expression of genes in Module 1 A), 4 B), 2 C), and 3 D) across the indicated genotypes. Each row corresponds

to an individual gene. Hierarchical clustering was performed based on dissimilarity of gene expression.

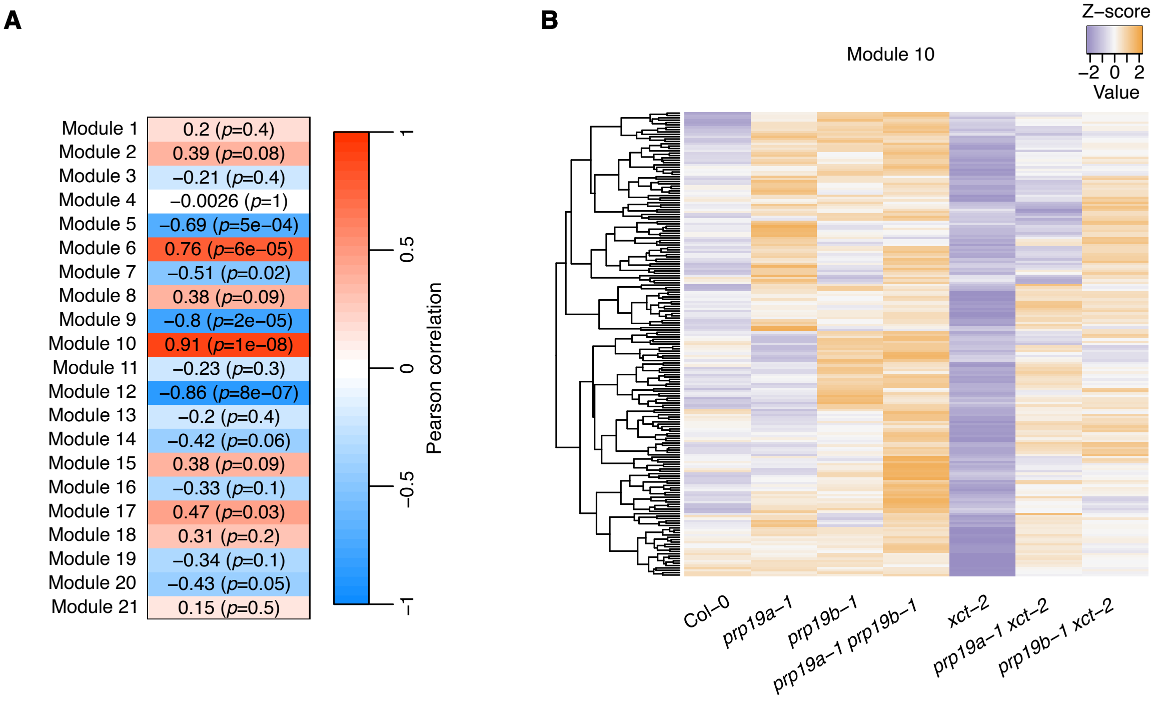

Supplementary Figure S10. Expression of Module 10 genes is positively correlated with circadian clock period length. A) Pearson correlation and the corresponding *p* value between circadian clock period length (shown in Figure 1A) and module eigengene expression (shown in Figure 4B) in Col-0, *prp19a-1*, *prp19b-1*, *prp19a-1 prp19b-1*, *xct-2*, *prp19a-1 xct-2* and *prp19b-1 xct-2*. Colors correspond to the values of Pearson correlation. B) Z-score transformed expression patterns of the 197 Module 10 genes across the indicated genotypes. Each row corresponds to an individual gene. Hierarchical clustering was performed based on dissimilarity of gene expression.

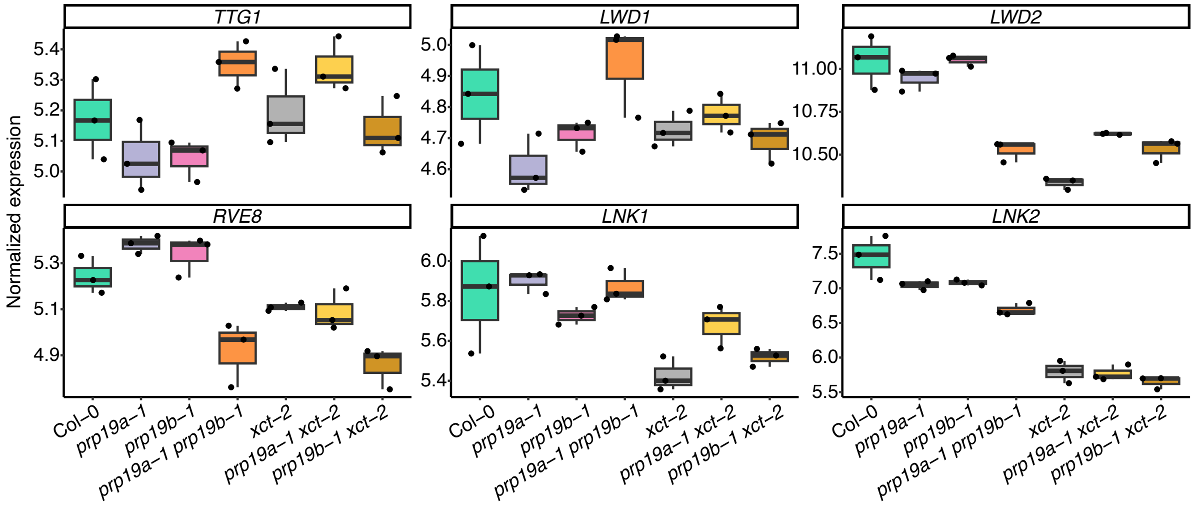

Supplementary Figure S11. Circadian clock genes known to be associated with anthocyanin biosynthesis are not antagonistically regulated by *XCT* and *PRP19*. Normalized RNA-seq read counts for *TTG1*, *LWD1*, *LWD2*, *RVE8*, *LNK1* and *LNK2* transcripts in the indicated genotypes. Each data point represents one biological replicate (n = 3). The upper edge, middle line, and lower edge in the boxplot represent the 75% quartile, median and 25% quartile of the data, respectively.

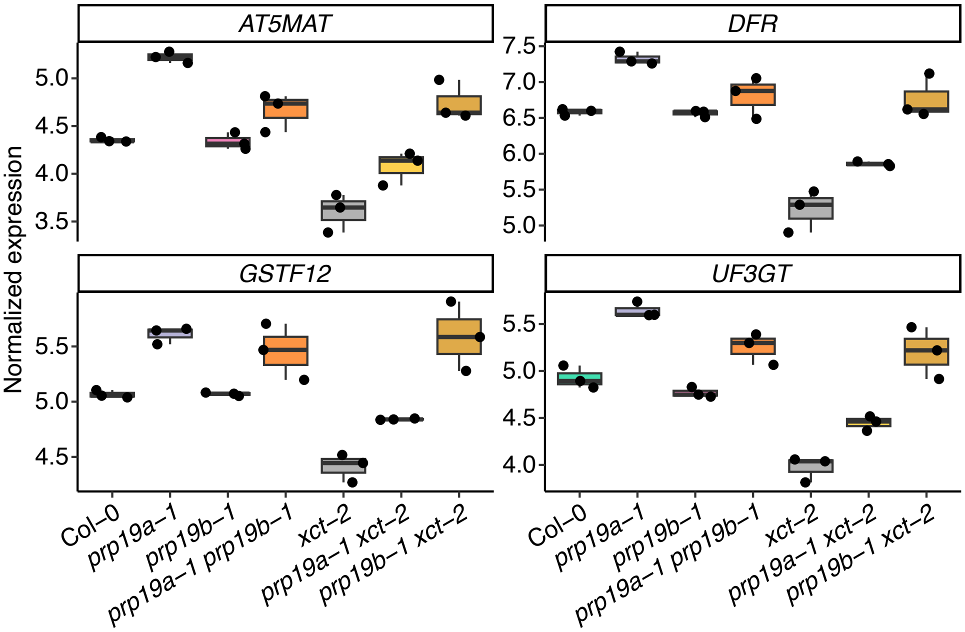

Supplementary Figure S12. Expression of genes encoding key anthocyanin biosynthetic enzymes is correlated with circadian clock period length. Normalized RNA-seq read counts for *AT5MAT*, *DFR*, *GSTF12*, and *UF3GT* transcripts in the indicated genotypes. Each data point represents one biological replicate (n = 3). The upper edge, middle line, and lower edge in the boxplot represent the 75% quartile, median and 25% quartile of the data, respectively.

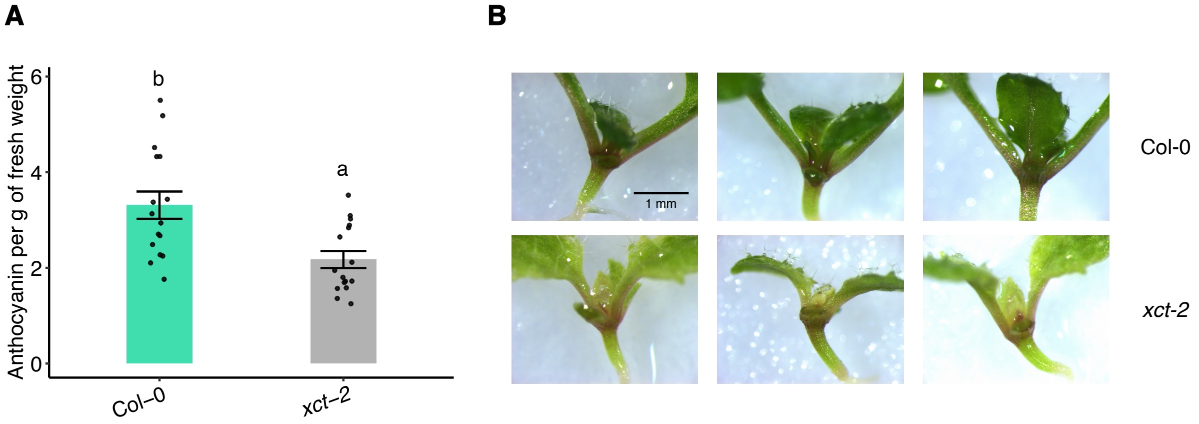

Supplementary Figure S13. Anthocyanin levels are significantly reduced in *xct-2* compared to Col-0. A) Quantification of anthocyanin levels in shoot tissue of 10-day-old Arabidopsis seedlings grown under constant white light. Bar plots represent means ± SE (n=16). Anthocyanin levels were calculated using the formula A530 – 0.25 x A657 and were normalized by fresh weight of the plant tissue. Letters indicate statistical significance determined by two-sided Welch's t-test ($p<0.01$). Experiments were independently repeated twice with similar results. B) Representative photos showing reduced anthocyanin level in the shoot apex and leaf petiole of *xct-2* compared to Col-0. Plants were grown in the same conditions as described in A). All panels in B) are shown at the same scale; the scale bar represents 1 mm.

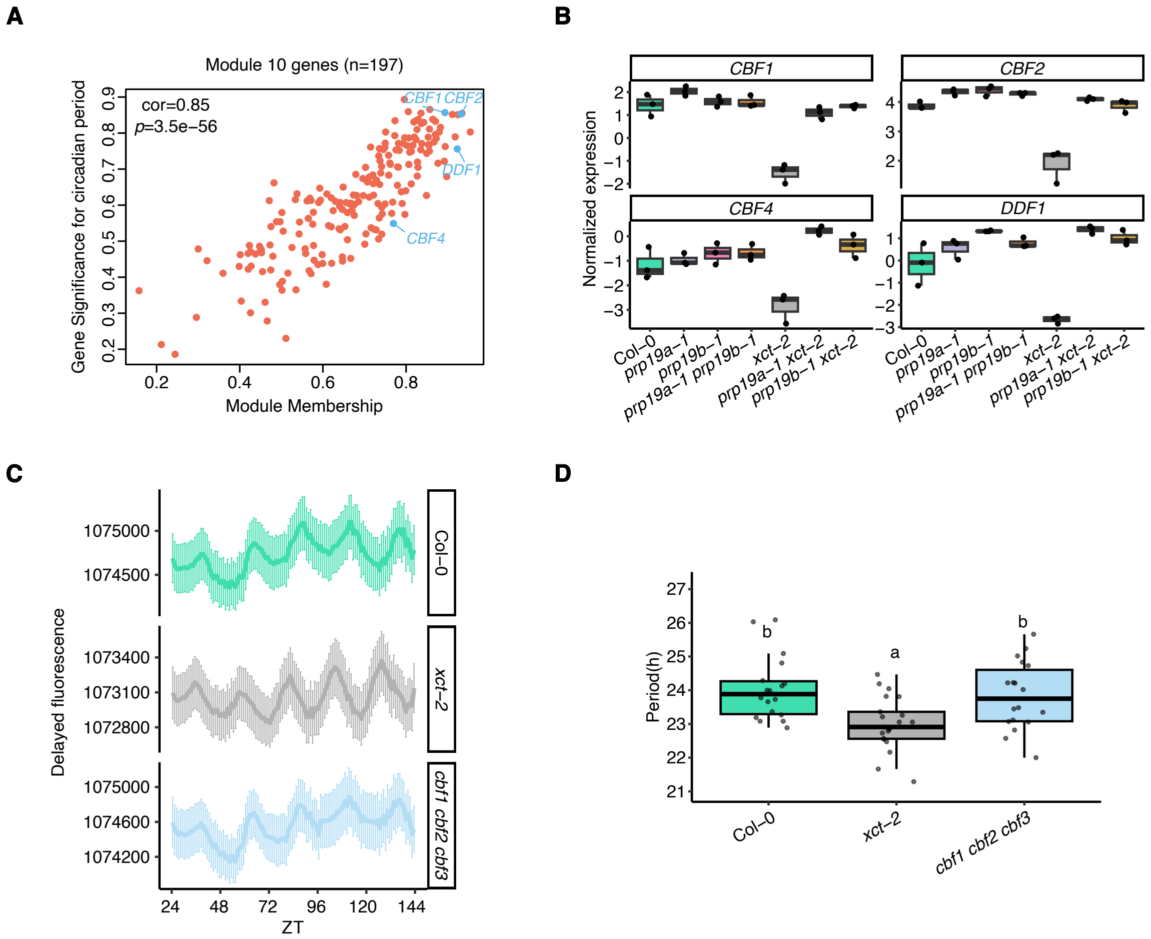

Supplementary Figure S14. ***CBF*** **family genes are regulated by XCT in a manner dependent on PRP19 function but do not impact circadian clock period.** A) Pearson correlation of module membership and gene significance values for the 197 Module 10 genes. Genes in *CBF* family are highlighted in blue. B) Normalized RNA-seq read counts for *CBF1*, *CBF2*, *CBF4*, and *DDF1* transcripts in the indicated genotypes. Each data point represents one biological replicate (n = 3). C) Delayed fluorescence rhythms in Col-0, *xct-2* and *cbf1 cbf2 cbf3* triple mutant seedlings. Traces represent mean ± SE (n=14-22) of detrended fluorescence intensity. 14-day-old plants entrained in 12-hour white light/12-hour dark were transferred at ZT0 to a chamber with constant temperature and light conditions for imaging. D) Circadian period of delayed fluorescence rhythms estimated from C). Statistical significance was determined using a linear regression model with genotype as a fixed effect and is indicated by

lower case letters (Tukey's multiple comparison test, $p < 0.05$). Experiments were independently repeated twice with similar results. The upper edge, middle line, and lower edge of the boxplots in B) and D) represent the 75% quartile, median and 25% quartile of the data, respectively.
